# *N*^6^-methyladenosine promotes induction of ADAR1-mediated A-to-I RNA editing to suppress aberrant antiviral innate immune responses

**DOI:** 10.1371/journal.pbio.3001292

**Published:** 2021-07-29

**Authors:** Hideki Terajima, Mijia Lu, Linda Zhang, Qi Cui, Yanhong Shi, Jianrong Li, Chuan He

**Affiliations:** 1 Department of Chemistry, Department of Biochemistry and Molecular Biology, and Institute for Biophysical Dynamics, The University of Chicago, Chicago, Illinois, United States of America; 2 Howard Hughes Medical Institute, The University of Chicago, Chicago, Illinois, United States of America; 3 Department of Veterinary Biosciences, College of Veterinary Medicine, The Ohio State University, Columbus, Ohio, United States of America; 4 Division of Stem Cell Biology Research, Department of Developmental and Stem Cell Biology, Beckman Research Institute of City of Hope, Duarte, California, United States of America; 5 Irell and Manella Graduate School of Biological Sciences, Beckman Research Institute of City of Hope, Duarte, California, United States of America; Yale University, UNITED STATES

## Abstract

Among over 150 distinct RNA modifications, *N*^6^-methyladenosine (m^6^A) and adenosine-to-inosine (A-to-I) RNA editing represent 2 of the most studied modifications on mammalian mRNAs. Although both modifications occur on adenosine residues, knowledge on potential functional crosstalk between these 2 modifications is still limited. Here, we show that the m^6^A modification promotes expression levels of the ADAR1, which encodes an A-to-I RNA editing enzyme, in response to interferon (IFN) stimulation. We reveal that YTH *N*^6^-methyladenosine RNA binding protein 1 (YTHDF1) mediates up-regulation of ADAR1; YTHDF1 is a reader protein that can preferentially bind m^6^A-modified transcripts and promote translation. Knockdown of YTHDF1 reduces the overall levels of IFN-induced A-to-I RNA editing, which consequently activates dsRNA-sensing pathway and increases expression of various IFN-stimulated genes. Physiologically, YTHDF1 deficiency inhibits virus replication in cells through regulating IFN responses. The A-to-I RNA editing activity of ADAR1 plays important roles in the YTHDF1-dependent IFN responses. Therefore, we uncover that m^6^A and YTHDF1 affect innate immune responses through modulating the ADAR1-mediated A-to-I RNA editing.

## Abstract 2

A study of the epitranscriptomic landscape reveals a functional role of the RNA modification N6-methyladenosine in suppressing aberrant innate antiviral immune responses by modulating A-to-I RNA editing and RNA secondary structure.

## Introduction

Posttranscriptional regulation plays a pivotal role in ensuring proper gene expression in almost all organisms. An emerging new mechanism of posttranscriptional regulation is conducted through various RNA modifications. With over 150 distinct chemical modifications known to exist in different RNA species [1], new scientific discoveries and technological advances have prompted rapid development of the field of epitranscriptomics [2,3]. Among mRNA modifications, 2 of the most well-characterized are *N*^6^-methyladenosine (m^6^A) and adenosine-to-inosine (A-to-I) RNA editing.

The profound impact of m^6^A modification on diverse biological functions and diseases have only recently been demonstrated [4,5]. The m^6^A modification exhibits biological functions through a series of m^6^A effector proteins generally referred to as writers, readers, and erasers, which deposit, recognize, and remove m^6^A, respectively. In mammals, the deposition of m^6^A on most mRNA is catalyzed by a large methyltransferase complex (writers) containing the core heterodimer of methyltransferase-like 3 (METTL3) and METTL14 [6–8]. m^6^A modification recruits m^6^A-binding proteins (readers) including several members of YTH family [9] that specifically recognize m^6^A to modulate various aspects of RNA metabolism. For example, YTH *N*^6^-methyladenosine RNA binding protein 1 (YTHDF1) promotes translation efficiency of m^6^A-modified mRNAs through recruiting translation initiation factors [10,11]. Another members of YTH family, YTHDF2, interacts with the CCR4-NOT deadenylase complex and facilitates degradation of its target mRNAs [12,13]. The removal of m^6^A is carried out by 2 demethylases (erasers), Fat mass and obesity-associated gene (FTO) [14], and alkB homolog 5 RNA demethylase (ALKBH5) [15]. The orchestration of writers and erasers functions suggests m^6^A as a reversible modification that can control dynamics of physiological processes [16].

On the other hand, A-to-I RNA editing is catalyzed by adenosine deaminase acting on RNA (ADAR) enzymes, ADAR1 and ADAR2, that specifically bind to double-stranded RNA (dsRNA) [17,18]. Among mammalian ADAR family members, editing activity of ADAR3 has not been detected. Because inosine base pairs preferentially with cytosine, A-to-I RNA editing can cause alteration in amino acid sequences [19], splicing [20], and dsRNA structures [21,22]. Recently, the ADAR1-mediated A-to-I RNA editing has emerged as a key regulatory factor that controls innate immune interferon (IFN) response [23,24]. Both *ADAR1* knockout mice and knock-in mice expressing the catalytic-deficient ADAR1 (ADAR1^E861A/E861A^) exhibited embryonic lethality with elevated IFN-stimulated genes (ISGs) signature and apoptosis [24,25]. The lethality can be rescued by concurrent knockout of *interferon induced with helicase C domain 1* (*IFIH1*, also called *MDA5*), a cytosolic sensor that recognizes viral dsRNA and induces the type I IFN-mediated antiviral immunity [25,26]. The molecular mechanism underlying these phenotypes is that ADAR1 unwinds dsRNA structure by RNA editing and prevents self-activation of the IFN response induced by endogenous dsRNA. Genomic mutations in *ADAR1* were identified in patients with Aicardi–Goutières syndrome (AGS), a severe autoimmune disease with a high IFN signature [27]. These studies indicated that the appropriate regulation of IFN signaling by ADAR1 is essential for normal homeostasis of the innate immune system.

Dysregulation in the expression of ADARs and A-to-I RNA editing is associated with a variety of physiological abnormalities including neuronal disorders [28,29], circadian rhythm disruption [30,31], and cancer progression [32]. Growing evidence shows a profound impact of ADAR1 on cancer immunotherapies through regulating IFN signaling. Loss of ADAR1 reduces cell viability in certain types of tumor cells expressing high levels of ISGs [33,34]. In addition, deletion of ADAR1 in tumor cells improves sensitivity to immunotherapy through enhancing IFN sensing and overcomes resistance to immune checkpoint blockage [35]. Therefore, a better insight into regulatory mechanisms of ADAR1 expression may provide further understanding to targeting ADAR1 in cancer therapies. Intriguingly, a recent study discovered a conserved m^6^A site in the *ADAR1* transcript among several primates [36], implying potential roles of m^6^A-mediated regulation of ADAR1 expression. However, biological functions of m^6^A on the *ADAR1* transcript have not been investigated. Here, we find that binding of YTHDF1 to m^6^A-modified mRNA enhances IFN-mediated ADAR1p150 induction. Knockdown of YTHDF1 attenuated global induction of A-to-I RNA editing upon IFN stimulation, resulting in activation of the dsRNA-sensing pathway. In the absence of YTHDF1, IFN stimulation causes enhanced innate immune responses, and virus replication in cells was suppressed by the elevated expression of ISGs. Our data uncover a new m^6^A-mediated regulation of A-to-I RNA editing and IFN response in innate immunity.

## Results

### m^6^A regulates interferon-induced expression of ADAR1p150

**Fig 1:**
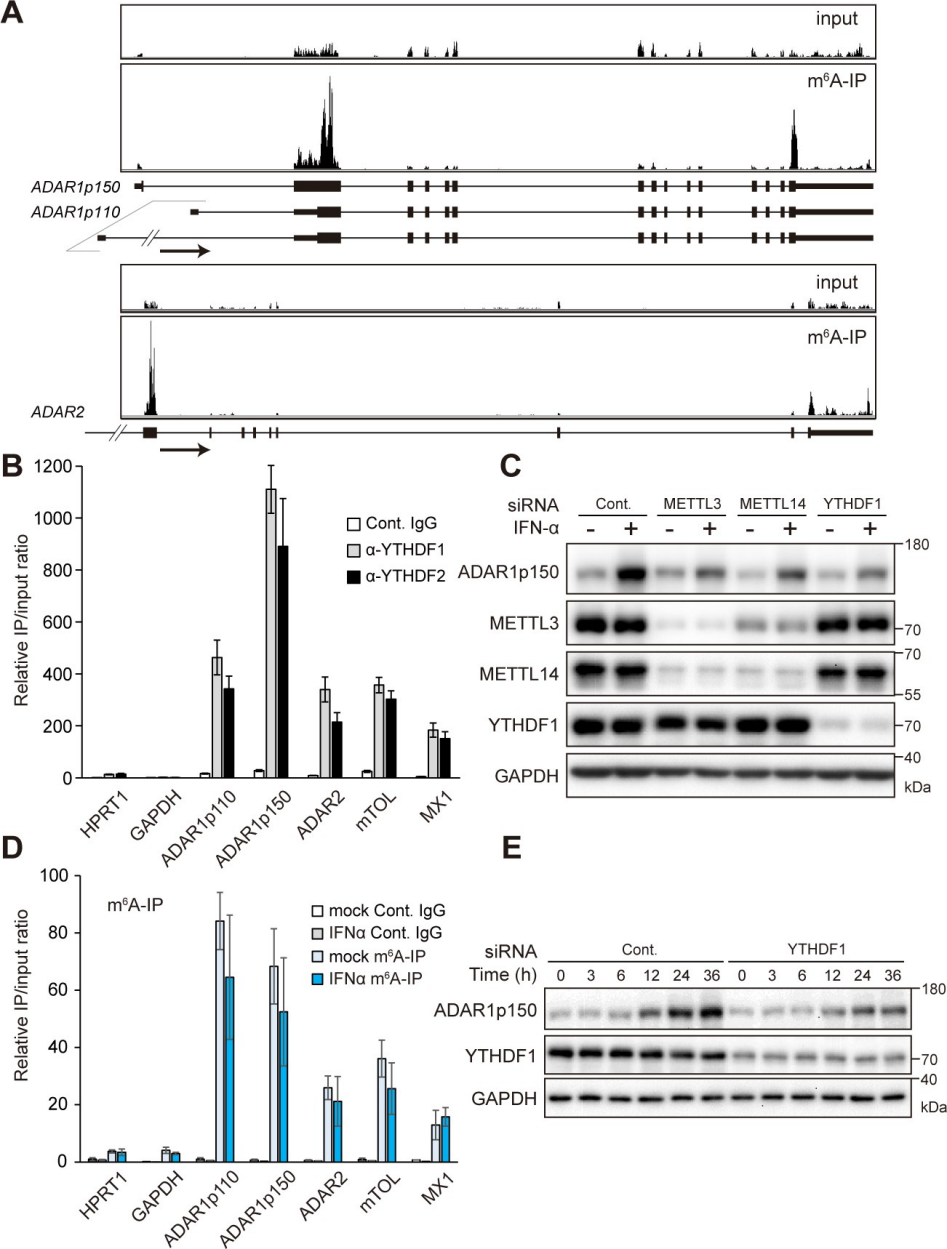
m^6^A methylation on *ADAR* mRNAs. (A) m^6^A-seq data of the *ADAR1* and *ADAR2* transcripts in HepG2 cells showing m^6^A modification on both transcripts, modified from Dominissini and colleagues [9]. An arrow indicates the transcription direction. (B) RIP RT-qPCR showing binding of YTHDF1 and YTHDF2 to *ADAR1p110* and *ADAR1p150* mRNAs in A172 cells. The signals were normalized to input samples. *HPRT1* and *GAPDH* were used as negative controls. *mTOL* and *MX1* were used as positive controls. (C) Immunoblot analysis showing knockdown effects of METTL3, METTL14, and YTHDF1 on IFN-induced ADAR1p150 protein expression in A172 cells. (D) m^6^A-RIP RT-qPCR showing m^6^A modification on *ADAR1p110* and *ADAR1p150* mRNAs. *HPRT1* and *GAPDH* were used as negative controls. *mTOL* and *MX1* were used as positive controls. (E) Immunoblot analysis showing time course of ADAR1p150 protein expression after IFN-α stimulation. Immunoblot images are representative of 3 biological replicates. (B, D) *n =* 3 for all experiments. Data are presented as the mean ± SEM. The numerical values for this figure are available in S1 Data. ADAR, adenosine deaminase acting on RNA; IFN, interferon; IgG, immunoglobulin G; IP, immunoprecipitation; m^6^A, *N*^6^-methyladenosine; RIP, RNA immunoprecipitation; RT-qPCR, quantitative reverse transcription PCR; SEM, standard error of the mean; siRNA, small interfering RNA.

A previous genome-wide analysis suggested a negative correlation between m^6^A methylation and A-to-I RNA editing, with depletion of m^6^A increasing editing [37]. In addition to this report, the observation of a conserved m^6^A site in the *ADAR1* transcript [36] prompted us to investigate functional connection between these 2 main types of mRNA modifications. The transcriptome-wide distributions of m^6^A in various cell types have been mapped using immunoprecipitation-based high-throughput sequencing approach [9,38]. We analyzed the previously published m^6^A-seq data and found that all of the 3 mammalian A-to-I RNA editing enzymes (ADAR1–3) have m^6^A sites in their mRNAs (Figs 1A and S1A). ADAR1 is expressed as 2 isoforms: a constitutively expressed 110-kDa isoform ADAR1p110, which primarily localizes to the nucleus, and an IFN-inducible 150-kDa isoform ADAR1p150, which localizes to both the cytoplasm and nucleus. RIP-qPCR with antibodies against YTHDF1 and YTHDF2 showed that both YTHDFs strongly bound to *ADAR1p110*, *ADAR1p150*, and *ADAR2* mRNAs in A172 glioblastoma cells, whereas ADAR3 was not expressed in A172 cells (Fig 1B). We next performed knockdown of m^6^A writers and readers and examined their effects on ADARs. Although METTL14 knockdown slightly reduced ADAR1p150 protein levels, knockdown of METTL3, YTHDF1, or YTHDF2 did not cause notable changes of both ADAR1p110 and ADAR1p150 protein expression levels in A172 cells under normal culture conditions (S1B and S1C Fig). On the other hand, YTHDF2 knockdown increased expression levels of both ADAR2 protein (S1B and S1C Fig) and mRNA (S1D Fig). YTHDF2 is known to facilitate mRNA degradation [12,13], suggesting that m^6^A methylation could suppress ADAR2 expression through controlling its transcript stability.

m^6^A modification enables rapid tuning of gene expression in response to cellular stress conditions such as heat shock [39,40] and oxidative stress [41]. Because IFN stimulation is known to induce *ADAR1p150* transcription from IFN-stimulated response element [42], we examined potential role of m^6^A on ADAR1p150 expression in response to human IFN-α stimulation. Immunoblot analysis revealed that knockdown of METTL3, METTL14, and YTHDF1 attenuated the IFN-mediated induction of ADAR1p150 (Fig 1C), whereas YTHDF2 knockdown did not noticeably alter ADAR1p150 protein expression upon IFN stimulation (S1E Fig). In contrast, the expression level of *ADAR1p150* mRNA was increased by knocking down METTL3 or YTHDF1 in IFN-treated cells (S1F Fig), which could be due to activation of upstream IFN pathway. The detailed mechanisms will be described in the latter part of this study. YTHDF1 promotes translation efficiency of target transcripts through interaction with translation initiation factors [10,43,44]. Our recent analysis indicated that depletion of YTHDF1 leads to an overall reduction of translation efficiency in transcripts harboring YTHDF1-bound m^6^A peaks [11]. Furthermore, we measured degradation rate of ADAR1p150 protein after treatment of cycloheximide (CHX) inhibiting new protein translation. YTHDF1 knockdown did not affect ADAR1p150 protein stability under the condition of IFN treatment (S1G Fig). The decreased ADAR1p150 protein level by IFN stimulation, despite the increased mRNA level and no change in the protein stability, clearly suggests that YTHDF1 promotes translation efficiency of *ADAR1p150* mRNA via m^6^A in IFN-treated cells (S1H Fig). Of note, m^6^A immunoprecipitation (m^6^A-IP) followed by quantitative reverse transcription PCR (RT-qPCR) revealed that the ratio of m^6^A-modified/total mRNA between mock and IFN-α treatment was not altered (Fig 1D). However, because IFN-α treatment increased the expression levels of both modified and unmodified *ADAR1p150* mRNA, more m^6^A-modified *ADAR1p150* transcripts were induced by IFN stimulation to produce more ADAR1p150 protein.

We next monitored the time course of the ADAR1p150 protein expression after IFN stimulation with YTHDF1 knockdown. We observed that the knockdown of YTHDF1 reduced and delayed the induction of the ADAR1p150 protein level in response to IFN (Figs 1E and S1I), indicating that YTHDF1 enables rapid expression of ADAR1p150 upon IFN stimulation.

### YTHDF1 affects A-to-I RNA editing levels

**Fig 2:**
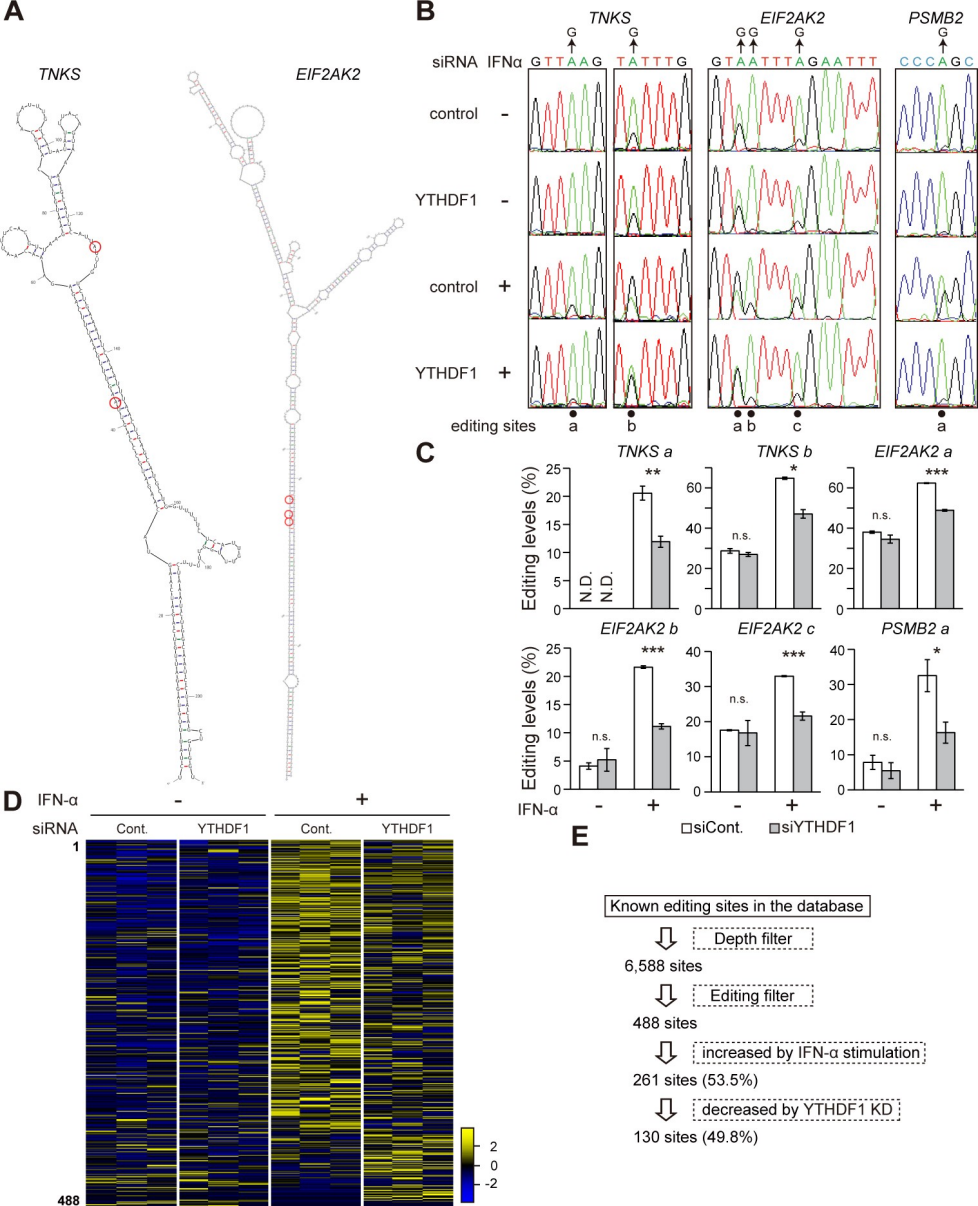
YTHDF1 knockdown reduces IFN-induced A-to-I RNA editing. (A) Predicted secondary structure of *TNKS* and *EIF2AK2* transcripts by the mfold web server. Red circles indicate A-to-I RNA editing sites. (B) Direct sequencing chromatogram showing from RT-PCR products of *TNKS*, *EIF2AK2*, and *PSMB2* mRNA following IFN-α stimulation. (C) Effect of YTHDF1 knockdown on A-to-I RNA editing of a few selected transcripts following IFN-α stimulation. Reduced IFN-induced A-to-I RNA editing was observed with YTHDF1 knockdown. Two-tailed Student *t* tests were performed to assess the statistical significance of differences between groups, *p < 0.05, **p < 0.01, ***p < 0.001, n.s. *p* ≧ 0.05, N.D. means not detected. *n =* 3 for all experiments. Data are presented as the mean ± SEM. (D) A heatmap of over editing levels from 3 biological replicates at 488 selected editing sites. Editing level changes were shown in each row covering all editing sites, ranked by the editing level. High editing levels were displayed in yellow and low in blue. An overall IFN-induced A-to-I RNA editing levels were reduced with YTHDF1 knockdown. (E) A flowchart for identification of IFN-induced editing sites. The numerical values for this figure are available in S1 Data. A-to-I RNA editing, adenosine-to-inosine RNA editing; IFN, interferon; KD, knockdown; n.s., not significant; RT-PCR, reverse transcription PCR; SEM, standard error of the mean; siRNA, small interfering RNA.

The effects of YTHDF1 on A-to-I RNA editing events were assessed in YTHDF1 knockdown cells. As inosine base pairs with cytosine, A-to-I RNA editing levels can be measured as an A-to-G conversion in the cDNA by reverse transcription (RT) and PCR. We quantified RNA editing levels [G / (A + G) (%)] at selected editing sites (Fig 2A) from direct sequencing chromatograms of the RT-PCR products (Fig 2B). In the transcripts coding *TNKS*, *EIF2AK2*, and *PSMB2*, A-to-I RNA editing levels were increased by IFN treatment due to the induction of ADAR1p150 (Fig 2B and 2C). YTHDF1 deficiency significantly reduced the IFN-mediated induction of editing levels at these sites (Fig 2B and 2C), which is consistent with the attenuation of ADAR1p150 induction by YTHDF1 knockdown (Fig 1C). To illustrate a comprehensive picture of IFN-induced changes in A-to-I RNA editing levels, we performed RNA-seq analysis in YTHDF1 knockdown cells with 3 biological replicates. For quantification of editing levels, the numbers of A and G bases at the known editing sites were counted from the aligned RNA-seq reads. Previous studies identified tens of thousands of A-to-I RNA editing sites in the human transcriptome, which can be found in the databases of REDIportal and RADAR [45,46]. Among these sites, 488 sites were edited in A172 glioblastoma cells according to our criteria (S2 Table), and about half of those editing sites (261 sites) showed more than 5% increase of the average editing levels after IFN stimulation (Fig 2D and 2E). About half of them (130 sites) exhibited more than 5% decrease of their average editing levels with YTHDF1 knockdown (Fig 2D and 2E), indicating that a large part of the IFN-induced editing events depend on the YTHDF1-mediated ADAR1p150 induction.

### YTHDF1 enhances interferon responses

**Fig 3:**
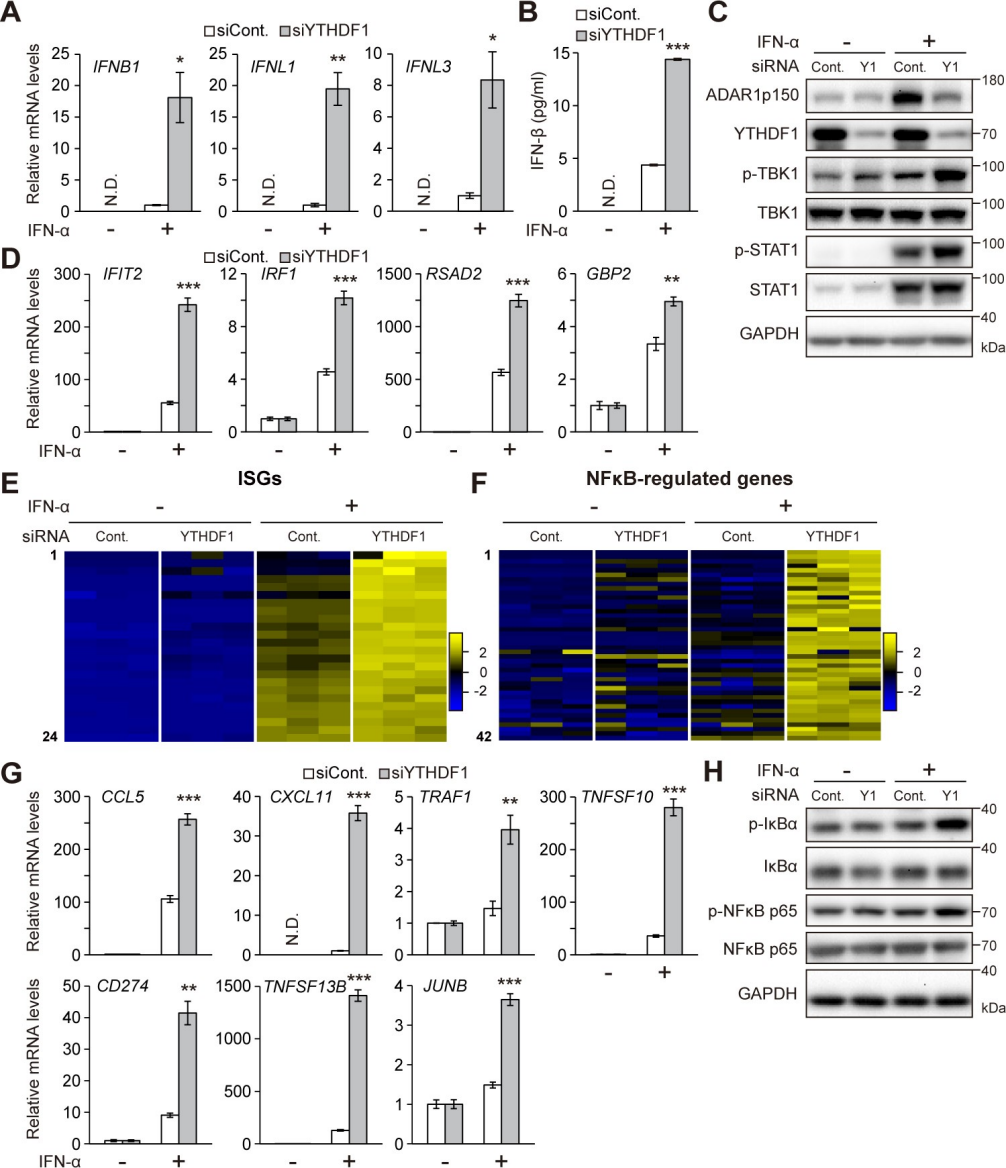
YTHDF1 knockdown induces IFN responses. Cells were treated with mock (−) or IFN-α (+). (A) RT-qPCR showing significantly elevated expression of IFN genes upon YTHDF1 knockdown and IFN stimulation. The signals were normalized to control siRNA samples. (B) Spontaneous IFN-β secretion by cells transfected with control or YTHDF1-specifc siRNA as quantified by ELISA after stimulation of IFN-α. (C) Immunoblot analyses of TBK1 and STAT1 phosphorylation levels. Immunoblot images are representative of 3 biological replicates. (D) RT-qPCR showing significantly elevated expression of ISGs upon YTHDF1 knockdown and IFN stimulation. The signals were normalized to mock treatment samples. (E, F) Heatmaps of expression levels of ISGs (E) and NF-κB–inducible genes (F) from RNA-seq data. (G) RT-qPCR showing significantly elevated expression of NF-κB–inducible genes upon YTHDF1 knockdown and IFN stimulation. The signals were normalized to mock treatment samples except for *CXCL11*. (H) Immunoblot analyzing of IκBα and NF-κB p65 phosphorylation levels. Immunoblot images are representative of 3 biological replicates. (A, D, G) The signals were normalized to *GAPDH*. (A, B, D, G) Two-tailed Student *t* tests were performed to assess the statistical significance of differences between groups, *p < 0.05, **p < 0.01, ***p < 0.001, N.D. means not detected. *n =* 3 for all experiments. Data are presented as the mean ± SEM. The numerical values for this figure are available in S1 Data. IFN, interferon; ISG, IFN-stimulated gene; RT-qPCR, quantitative reverse transcription PCR; SEM, standard error of the mean; siRNA, small interfering RNA; TBK1, TANK-binding kinase 1.

Recent studies have shown that ADAR1-mediated A-to-I RNA editing disrupts secondary structures of dsRNA to prevent self-activation of cytosolic viral dsRNA sensors that induce IFN production and downstream ISGs, contributing to antiviral immunity [23]. These observations prompted us to explore potential roles of YTHDF1 in the IFN signaling. YTHDF1 knockdown significantly enhanced the induction of not only *interferon beta 1* (*IFNB1*) but also *interferon lambda 1* (*IFNL1*) and *IFNL3* mRNAs following stimulation with IFN-α (Fig 3A). Consistent with this, the IFN-α–induced secretion of the IFN-β protein was higher in YTHDF1-deficient cells than that in control cells, whereas IFN-β was not detected in unstimulated cells (Fig 3B). *IFNB1* mRNA is known to be m^6^A modified, and its stability could be affected by m^6^A in certain cells [47,48]. However, decay rates of *IFNB1* and *IFNL1* transcripts were not altered with YTHDF1 knockdown in A172 cells (S2A Fig). Thus, the up-regulation of IFN-β is not caused by the m^6^A-dependent regulation of the *IFNB1* mRNA stability, but rather by signaling pathways upstream of *IFNB1* transcription in this cell line. During dsRNA-sensing response, the transcription of *IFNB1* is under the control of the transcription factor *IFN regulatory factor 3* (*IRF3*) that is activated by *TANK-binding kinase 1* (*TBK1*). We found that phosphorylation of TBK1, a hallmark of TBK1 activation, was drastically increased by YTHDF1 knockdown following stimulation with IFN-α (Figs 3C and S2B), demonstrating that YTHDF1 depletion could activate dsRNA-sensing pathway that induces *IFNB1* transcription.

The type I IFNs including IFN-α and IFN-β trigger STAT1 phosphorylation and subsequently initiate transcription of various ISGs. Our RT-qPCR analysis and RNA-seq data revealed that the expression levels of ISGs transcripts were highly induced by IFN-α treatment in YTHDF1 knockdown cells (Fig 3D and 3E, S3 Table), as a result of high IFN-β secretion. As expected, enhanced induction of STAT1 phosphorylation by IFN-α treatment was observed in YTHDF1 knockdown cells (Figs 3C and S2B). The cytosolic dsRNA-sensing pathway also causes activation of NF-κB, which induces transcription of a variety of genes involved in immune and inflammatory responses. We found that the expression levels of several NF-κB–regulated genes were increased by YTHDF1 knockdown with IFN-α stimulation in our RNA-seq data (Fig 3F and S3 Table) and RT-qPCR analysis (Fig 3G). The transcriptional activation of NF-κB is regulated by its interaction with inhibitory modulator IκBα that retains NF-κB in the cytoplasm. In response to various pathways including dsRNA signaling, IκBα is phosphorylated and degraded via the ubiquitin–proteasome system. Phosphorylation of NF-κB is also known to contribute to its activation. The phosphorylation of IκBα and NF-κB were increased in YTHDF1 knockdown cells following stimulation with IFN-α (Figs 3H and S2B), indicating that NF-κB signaling pathway is also activated by YTHDF1 deficiency under the condition of IFN treatment.

### Enzymatic activity of ADAR1 is required to enhanced interferon responses in YTHDF1-deficient cells

**Fig 4:**
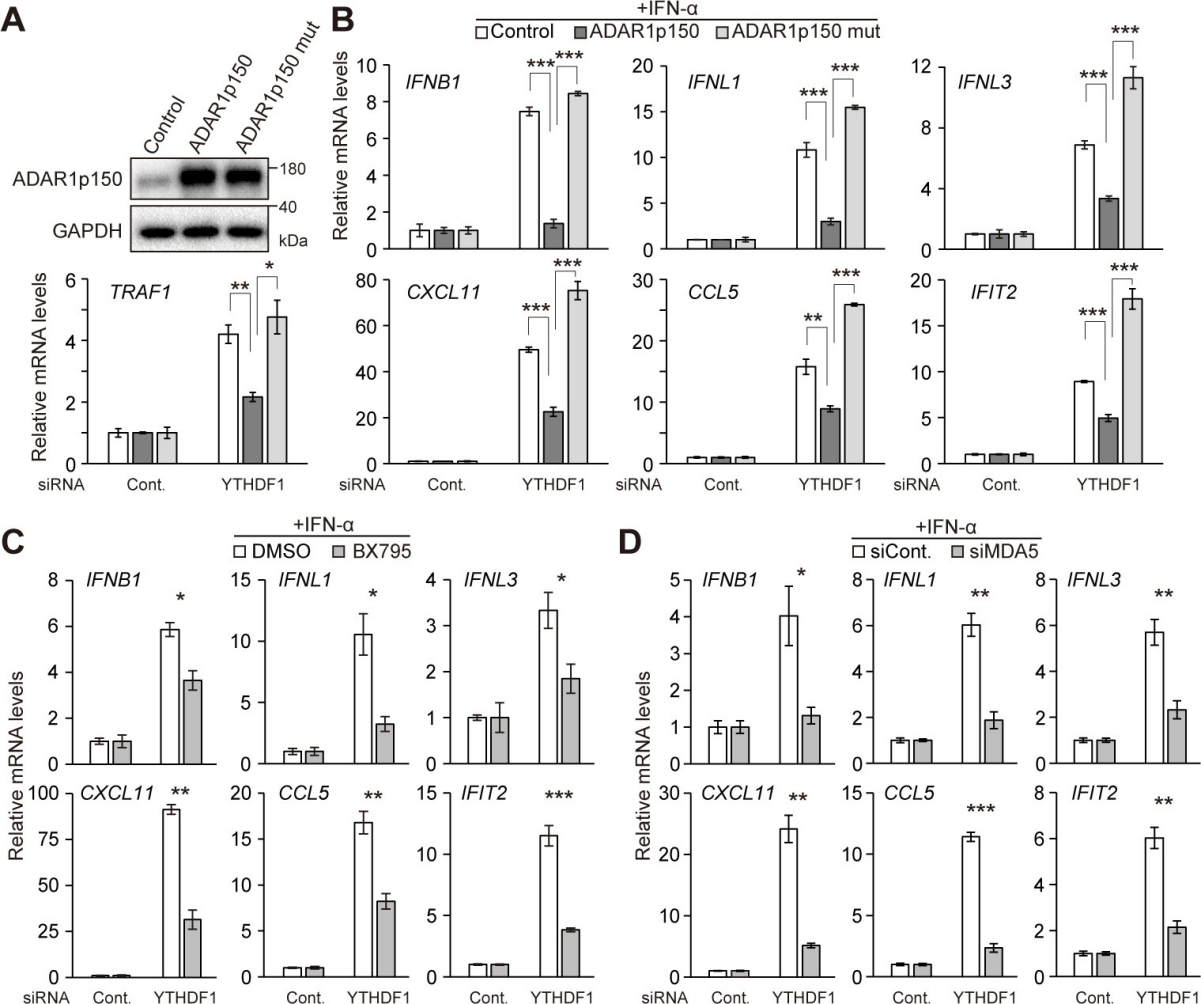
ADAR1p150 A-to-I RNA editing activity is required for the YTHDF1-dependent IFN responses. (A) Immunoblot analysis in stable cell lines with lentivirus expressing control EGFP, wild-type ADAR1p150, or catalytically inactive mutant of ADAR1p150^E912A^, respectively. (B) RT-qPCR showing knockdown effect of YTHDF1 on IFN genes, ISGs, and NF-κB–inducible genes in stable cell lines following IFN-α stimulation. The enhanced IFN responses were attenuated by overexpression of ADAR1p150, but not by catalytic inactive mutant ADAR1p150^E912A^. (C) RT-qPCR showing knockdown effect of YTHDF1 on IFN genes, ISGs, and NF-κB–inducible genes in A172 cells that were pretreated with the BX759 inhibitor for 1 h and then treated with IFN-α. The enhanced IFN responses were attenuated by the BX759. (D) RT-qPCR showing that concomitant knockdown of YTHDF1 and MDA5 attenuated the enhanced expression of IFN genes, ISGs, and NF-κB–inducible genes upon IFN-α stimulation. (B–D) The signals were normalized to *GAPDH* and then normalized to control siRNA samples. Two-tailed Student *t* tests were performed to assess the statistical significance of differences between groups, *p < 0.05, **p < 0.01, ***p < 0.001. *n =* 3 for all experiments. Data are presented as the mean ± SEM. The numerical values for this figure are available in S1 Data. A-to-I RNA editing, adenosine-to-inosine RNA editing; IFN, interferon; ISG, IFN-stimulated gene; RT-qPCR, quantitative reverse transcription PCR; SEM, standard error of the mean; siRNA, small interfering RNA.

To test whether the enhanced IFN responses in YTHDF1 knockdown cells were caused by the reduction of ADAR1p150 expression, we generated stable A172 cell lines that express control EGFP, ADAR1p150, and catalytically inactive mutant of ADAR1p150^E912A^, which is previously established [49] (Fig 4A), respectively. Overexpression of wild-type ADAR1p150 exhibited significantly enhanced A-to-I RNA editing activity, while overexpression of ADAR1p150^E912A^ did not increase editing activity (S2C Fig). Strikingly, the enhanced *IFNB1* induction was completely abolished by overexpression of ADAR1p150 (Fig 4B). Similarly, overexpression of ADAR1p150 suppressed *IFNL1*, *IFNL3*, and other ISGs following stimulation with IFN (Fig 4B). These inhibitory effects were abrogated by overexpression of a catalytic inactive mutant ADAR1p150^E912A^ (Fig 4B). These data indicate that the aberrant activation of IFN pathways by YTHDF1 knockdown following IFN treatment is largely due to the defects of ADAR1p150 induction and its A-to-I RNA editing activity.

Furthermore, we investigated the contribution of downstream components of ADAR1-regulated signaling pathway. It is known that ADAR1 destabilizes secondary structure of endogenous RNA to prevent self-activation of the dsRNA sensors, retinoic acid–inducible gene I (RIG-I)–like receptors (RLRs) such as MDA5 [23]. RLR activation recruits and activates mitochondrial antiviral-signaling protein (MAVS), leading to phosphorylation of TBK1 and downstream transcription of IFN genes. To examine the effect of this signaling pathway, we used a TBK1 inhibitor (BX795) that effectively inhibits the induction of IFNs genes upon stimulation with synthetic analog of dsRNA, poly (I:C), in A172 cells (S2D Fig). Pretreatment of YTHDF1 knockdown cells with BX795 attenuated the induction of IFNs genes and downstream ISGs expression following IFN-α stimulation (Fig 4C). In addition, concomitant knockdown of MDA5 and YTHDF1 markedly suppressed the induction of IFNs genes and ISGs (Figs 4D and S2E). Taken together, our results indicate that the YTHDF1-mediated induction of ADAR1p150 and its editing activity are critical for preventing undesirable activation of MDA5, which could cause phosphorylation of TBK1 and excessive downstream IFN production during IFN response.

### YTHDF1 affects cellular interferon-inducible cell growth and apoptosis

To elucidate the role of YTHDF1 in cellular response to IFN stimulation, YTHDF1 was stably knocked down by short hairpin RNA (shRNA) in A172 cells. The established cell line showed reduced ADAR1p150 induction and enhanced gene expression of IFNs in response to IFN-α as same phenotypes as those in transient knockdown cells (S3A and S3B Fig). IFN-α treatment reduced cell proliferation rates of the YTHDF1 knockdown cells, whereas mock treatment showed no noticeable differences in cell proliferation between the control cells and the YTHDF1-deficient cells (S3C Fig). Consistent with the previous observation (Fig 4B), overexpression of wild-type ADAR1p150 partially attenuated the reduced cell proliferation upon YTHDF1 knockdown compared with the control cells (S3C Fig). The effect was not observed by overexpression of ADAR1p150^E912A^ (S3C Fig), suggesting that A-to-I RNA editing activity of ADAR1p150 are partially responsible for the YTHDF1 knockdown effect on cell proliferation following IFN treatment. In addition, stable knockdown of MDA5 (S3D Fig) also attenuated the inhibitory effect on cell proliferation (S3E Fig). Meanwhile, the YTHDF1-deficient cells were more susceptible to apoptosis induction by IFN than were the control cells (S3F Fig). MDA5 knockdown diminished the elevated apoptosis signals in YTHDF1 knockdown cells (S3G Fig), suggesting the importance of the dsRNA-sensing pathway in these YTHDF1 function. These results demonstrate that the enhanced IFN responses in YTHDF1 knockdown cells upon IFN stimulation decrease cellular growth and increase apoptosis.

### Cell type–specific YTHDF1 knockdown effects

We also investigated whether YTHDF1 regulates ADAR1p150 expression and IFN responses in other cell lines that include LN229 glioblastoma, HeLa, and HEK293T. Immunoblot analysis revealed that knockdown of YTHDF1 attenuated the IFN-mediated induction of ADAR1p150 protein upon IFN stimulation in LN229 and HeLa cells (S4A Fig). The expression level of *ADAR1p150* mRNA was not changed by knocking down YTHDF1 in both mock and IFN-treated cells (S4B Fig). These data suggest that YTHDF1 promotes translation efficiency of *ADAR1p150* mRNA in IFN-treated LN229 and HeLa cells, which is consistent with our findings in A172 cells. In contrast, this YTHDF1 effect on ADAR1p150 expression was not observed in HEK293T cells (S4A Fig). In addition, we have examined IFN responses in these cell lines with YTHDF1 knockdown. In LN229 cells, YTHDF1 knockdown significantly enhanced the induction of IFNs, ISGs, and NF-κB-regulated genes following stimulation with IFN-α (S4C Fig), as observed in A172 cells. IFN-α treatment reduced cell proliferation rate and increased apoptosis in stable YTHDF1 knockdown LN229 cells (S4D–S4F Fig). On the other hand, IFNs genes were not expressed in HeLa and HEK293T cells even though treated with IFN-α, suggesting that YTHDF1 does not affect IFN pathway in HeLa and 293T cells. Taken together, our results imply the presence of a cell type–specific mechanism that YTHDF1 promotes IFN-mediated ADAR1p150 induction and regulates IFN responses. Although the reason that only A172 and LN229 glioblastoma cells exhibit this regulation remain elusive, it might be possible that these differences are due to cell context differences such as expression levels of YTHDF1 protein, m^6^A modification levels of *ADAR1p150* mRNA, or sensitivity to dsRNA detection.

### YTHDF1 knockdown inhibits viral replication in cells

**Fig 5:**
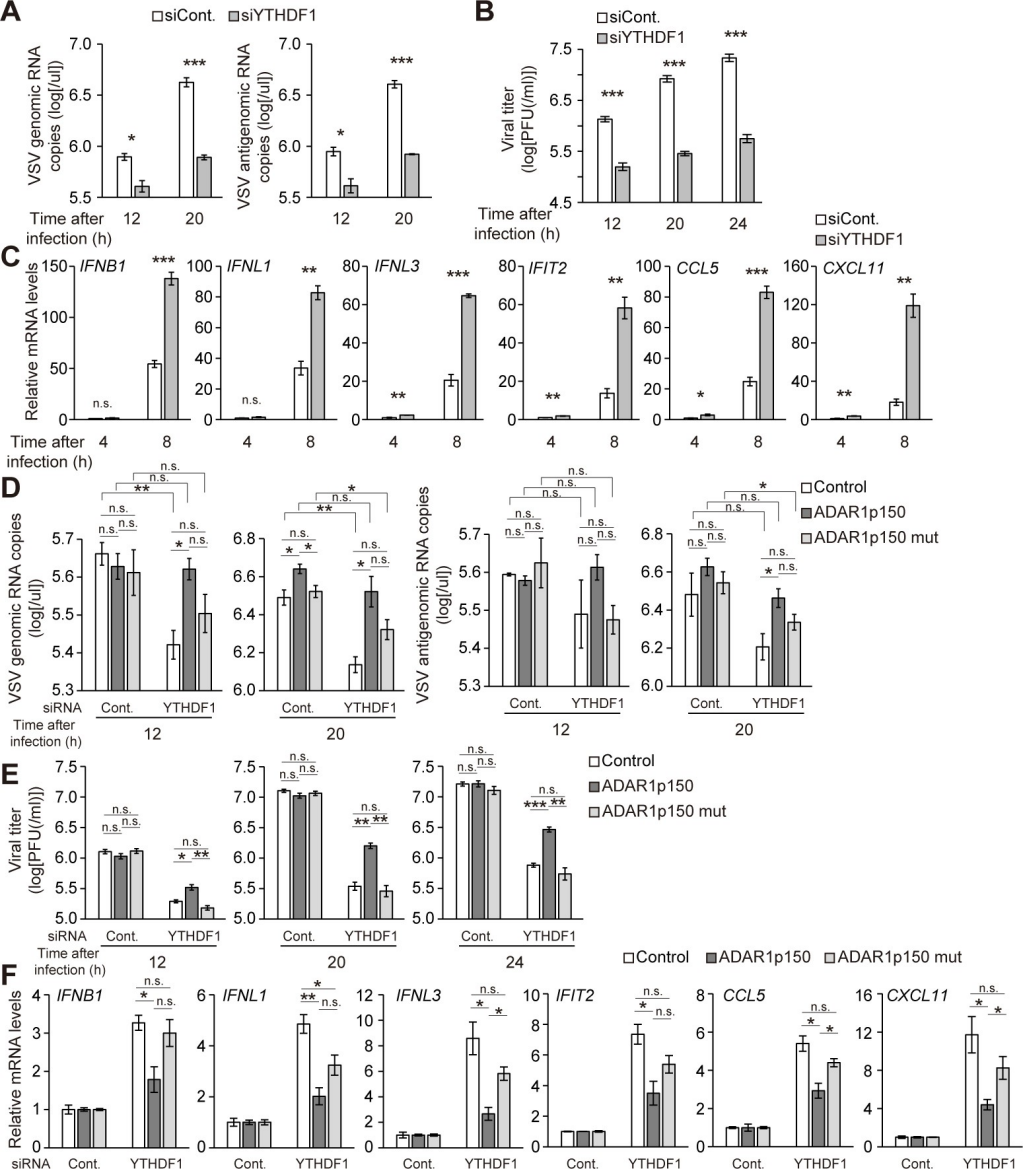
YTHDF1 knockdown inhibits viral replication in cells. (A) RT-qPCR showing significant decrease in the expression of VSV genome RNA and antigenome RNA upon YTHDF1 knockdown at 12 and 20 h after rVSV-GFP infection. (B) Viral titers in culture medium at 12, 20, and 24 h after rVSV-GFP infection were determined by plaque assay. YTHDF1 knockdown inhibited the release of infectious virus. (C) RT-qPCR showing elevated expression levels of IFN genes, ISGs, and NF-κB–inducible genes upon YTHDF1 knockdown at 4 and 8 h after rVSV-GFP infection. The signals were normalized to *GAPDH*. (D) RT-qPCR showing knockdown effect of YTHDF1 on the expression of VSV genome RNA and antigenome RNA at 12 and 20 h after rVSV-GFP infection in stable cell lines. (E) Viral titers in culture medium at 12, 20, and 24 h after rVSV-GFP infection were determined by plaque assay. (F) RT-qPCR showing knockdown effect of YTHDF1 on IFN genes, ISGs, and NF-κB–inducible genes in stable cell lines. The signals were normalized to *GAPDH* and then normalized to control siRNA samples. (A–F) Two-tailed Student *t* tests were performed to assess the statistical significance of differences between groups, *p < 0.05, **p < 0.01, ***p < 0.001, n.s. *p* ≧ 0.05. *n =* 3 for all experiments. Data are presented as the mean ± SEM. The numerical values for this figure are available in S1 Data. IFN, interferon; ISG, IFN-stimulated gene; n.s., not significant; RT-qPCR, quantitative reverse transcription PCR; rVSV-GFP, recombinant GFP-expressing vesicular stomatitis virus; SEM, standard error of the mean; siRNA, small interfering RNA.

We next investigated a YTHDF1 knockdown effect on viral replication in A172 cells to demonstrate a physiological role of YTHDF1 in antiviral activities of IFN pathway. YTHDF1 knockdown cells were infected with recombinant GFP-expressing vesicular stomatitis virus (rVSV-GFP), a prototypic nonsegmented negative-stranded RNA virus whose replication is highly sensitive to the antiviral activities of IFN. We found that GFP expression was markedly reduced in YTHDF1 knockdown cells (S5A Fig). This reduction in viral replication was also observed in the decreased expression levels of viral G protein (S5B and S5C Fig), viral genome and the replicative intermediate antigenome RNA (Fig 5A), and the release of infectious virus (Fig 5B). YTHDF1 knockdown significantly increased the expression levels of IFN genes, ISGs, and NF-κB–regulated genes at early time points after VSV infection (Fig 5C). These results indicated that YTHDF1 depletion enhances cellular antiviral activities through increasing IFN responses during viral infection.

We also examined VSV replication in stable cell lines expressing ADAR1p150 or ADAR1p150^E912A^ upon YTHDF1 knockdown to investigate contributions of ADAR1 and A-to-I RNA editing activity to this antiviral activity. Overexpression of ADAR1p150 significantly attenuated the YTHDF1-mediated suppression of viral genome RNA expression (Fig 5D) and the release of infectious virus (Fig E). In addition, overexpression of ADAR1p150 slightly recovered the reduced expression levels of viral G protein caused by YTHDF1 knockdown, though the effect was marginal and observed only at 12 h after VSV infection (S5D and S5E Fig). It is possible that YTHDF1 can directly affect viral protein expression because a very recent study showed that VSV viral transcripts have m^6^A sites [50], and our study revealed that YTHDF proteins affect the expression levels of viral G protein through binding to m^6^A sites in their mRNA [51]. The enhanced mRNA expression of IFN genes, ISGs, and NF-κB–regulated genes were partially suppressed by ADAR1p150 overexpression (Fig 5F), which is consistent with the increased viral replication in ADAR1p150-overexpressing cell lines. These effects were not observed or diminished by overexpression of mutant ADAR1p150^E912A^ (Figs 5D–5F and S5D and S5E). Our results demonstrate ADAR1p150 and its A-to-I RNA editing activity as a significant mediator for proviral function of YTHDF1.

Collectively, m^6^A methylation and the m^6^A reader YTHDF1 promote expression levels of IFN-induced ADAR1p150 and regulate A-to-I RNA editing in response to IFN. Several IFN-induced transcripts possess A-to-I RNA editing sites in their double-stranded region (Figs 2B and S6A–S6C), which should affect dsRNA structure. In fact, flow cytometry analysis with dsRNA-specific J2 antibody staining showed that IFN stimulation increased the number of dsRNA positive cells and YTHDF1 knockdown enhanced the IFN-mediated induction of dsRNAs (S6D Fig). The YTHDF1-mediated ADAR1p150 induction can prevent accumulation of unedited dsRNAs to suppress activation of MDA5 and consequent excessive IFN response (S6E Fig). During viral infection, loss of YTHDF1 exhibited the antiviral activities following with the enhanced IFN responses.

## Discussion

IFN family members are central regulatory cytokines associated with protective immune response to infection, whereas an aberrant activation of IFN pathways cause autoimmune diseases such as AGS. Therefore, fine-tuned production of IFNs and balanced activation of the subsequent responsive genes are necessary for proper physiological activity. The present study showed that YTHDF1 is an important suppressor of IFN responses, which may prevent cells from the harmful effects of overactivated IFN signaling and downstream ISGs. YTHDF1 promotes expression levels of ADAR1p150 whose mRNA is m^6^A modified and is induced by IFN stimulation. Some IFN-induced transcripts have A-to-I RNA editing sites, indicating the presence of double-stranded structure in these transcripts (Figs 2B and S6A–S6C). Although we have not yet clarified key dsRNA-containing transcripts, which are responsible for MDA5 recognition, these IFN-inducible dsRNAs can be potential activator for dsRNA-sensing pathway, resulting in positive feedback of IFN responses. Our data revealed that the ADAR1-mediated A-to-I RNA editing, facilitated by the YTHDF1-mediated up-regulation, is important for inhibiting this feedback and preventing excessive activation of IFN responses. Therefore, functional regulation of YTHDF1 may serve to a promising therapeutic strategy for autoimmune diseases associated with high IFN signatures.

Translation up-regulation by YTHDF1 has been confirmed by various groups in diverse biological systems [10,43,44,52–55]. It can be induced by stimulation as we have shown recently [43]. A recent study has questioned whether YTHDF1 affects translation of m^6^A-modified mRNA in HeLa cells [56]. However, the cumulative fraction analysis from the overall ribosome profiling data would have missed YTHDF1 regulation on its individual targets. Indeed, our recent QTL analysis revealed that depletion of m^6^A causes not only reduction but also increase of translation efficiency in transcripts harboring m^6^A sites [11]. Translation regulation of m^6^A-modified mRNA can be heterogeneous in HeLa cells; however, translation up-regulation of YTHDF1-bound transcript in HeLa cells is clear [10].

One of the important physiological functions of IFN is the antiviral immune responses to viral infection. Recent studies indicated that m^6^A modification can play either proviral [47,48,50,51,57] or antiviral [44,58] roles in virus life cycle. A series of m^6^A writers, readers and erasers were increased upon human cytomegalovirus (HCMV) infection [47,48]. In A172 cells examined in this current study, the protein expression levels of METTL3, METTL14, YTHDF1, and YTHDF2 were not affected by IFN-α stimulation (Figs 1C and S1E), suggesting that the induction of m^6^A effectors may not be caused by virus-induced type I IFN pathways in A172 cells. In addition, several papers showed that the expression levels of not only *IFNB1* [47,48] but also various ISGs [44] are regulated by the m^6^A-mediated mechanisms. Based on the results that the antiviral effects of YTHDF1 knockdown were not completely recovered by overexpression of ADAR1p150 (Fig 5D and 5E), there could be other m^6^A-regulated ISGs that are responsible for the effects of YTHDF1 knockdown upon viral infection. It should be noted that YTHDF1 does not directly affect *IFNB1* mRNA stability in A172 cells (S2A Fig). We also demonstrated that A172 and LN229 glioblastoma cells showed YTHDF1-mediated suppression of IFN responses, while HeLa and 293T cells did not (S4A–S4C Fig). We believe that these differences are due to cell context differences such as expression levels of YTHDF1 protein, m^6^A modification levels of *ADAR1p150* mRNA, or sensitivity to dsRNA detection. In fact, our ongoing work does suggest that the relative expression levels of YTHDF1 and other RNA-binding proteins critically impact translation regulation through YTHDF1, which we will report in the future.

A recent study found that m^6^A modification negatively regulates A-to-I RNA editing through inhibiting the association of m^6^A-modified mRNAs with ADAR enzymes [37]. They showed that knockdown of m^6^A writers did not affect expression of ADAR enzymes under normal cellular conditions. We revealed that, upon IFN stimulation, m^6^A appears to affect ADAR1p150 translation and A-to-I RNA editing. We have previously proposed that YTHDF1 may enable generation of a pulse of protein synthesis through translation promotion [4]. The present study suggested a possible mechanism that this type of m^6^A-mediated regulation on A-to-I RNA editing restricts the extent and length of the IFN responses to balance toxic effects on cells during viral infection or IFN stimulation. Interestingly, the m^6^A regulation of ADAR1p150 expression does not seem to be facilitated by YTHDF2 upon IFN stimulation (S1E Fig). In contrast, we observed that YTHDF2 affects ADAR2 expression but not ADAR1, though its physiological role remains to be investigated in the future. We currently do not have any clue about how YTHDF proteins achieve selectivity of functions among transcripts. While m^6^A could affect A-to-I RNA editing, an examination of the A-to-I RNA editing databases REDIportal and RADAR [45,46] also revealed numerous RNA editing sites in the transcripts encoding m^6^A-related genes such as METTL3, METTL16, YTHDF3, YTHDC1, YTHDC2, and ALKBH5. A-to-I RNA editing is known to regulate expression, stability, and alternative splicing of target mRNA, suggesting that it is possible that ADAR enzymes could conversely affect m^6^A methylation through modifying these m^6^A-related transcripts. Crosstalk between A-to-I RNA editing and m^6^A methylation might be more complicated, in particular upon immune stimulation or other stress responses.

The IFN family members play an important role in not only antiviral innate immunity but also cell proliferation, adaptive immunity, and cancer therapy [59]. In certain types of cancers, elevated ADAR1 expression and consequent hyperediting are caused by enhanced IFN signatures due to inflammatory environment of cancers, which drives cancer progression [60]. Several recent reports found that tumor cells exhibit a vulnerability to ADAR1 deficiency [33,34]. Loss of ADAR1 overcomes a resistance to a checkpoint that normally reduces sensing of endogenous dsRNAs, leading to IFN-mediated inflammation and increase in the efficacy of immunotherapy [35]. Furthermore, Our previous report found that YTHDF1 suppresses CD8^+^ T cell antitumor response through controlling cross-presentation of tumor antigens [55]. These reports and the present study together suggest that developing selective inhibitors of YTHDF1 function may also be immunotherapy strategies for certain tumors through enhancing IFN responses in tumor cells.

## Methods

### Cell lines, antibodies, and siRNA knockdown

The A172 (CRL-1620) and LN229 (CRL-2611) cells were purchased from ATCC and maintained in Dulbecco’s modified Eagle’s medium (DMEM; Gibco, 11965) supplemented with 10% fetal bovine serum (FBS, Gibco) and 1% penicillin–streptomycin (Gibco, 15140) at 37°C in 5% CO_2_. The cells expressing the control EGFP (pLJM1-EGFP), ADAR1p150, mutant ADAR1p150, control shRNA, or shRNA targeting YTHDF1 were maintained in DMEM, 10% FBS, 1% penicillin–streptomycin, and 1 μg/ml of puromycin for selection. The primary antibodies were purchased from commercial sources, and information about the antibodies was given in S1 Table. Actinomycin D (A9415) was purchased from Sigma, recombinant human IFN-α (11200) was purchased from PBL Assay Science, and BX795 (tlrl-bx7) was purchased from Invivogen. siRNAs against METTL3, METTL14, YTHDF1, YTHDF2, and MDA5 were purchased from SIGMA. The sequences of siRNAs were summarized in S1 Table. AllStars negative control siRNA (QIAGEN, 1027281) was used as control siRNA in knockdown experiments. Cells were transfected by using the Lipofectamine RNAiMAX (Invitrogen) for siRNAs and PEI-MAX (Polysciences, 24765) for plasmids according to the manufacturer’s protocols, respectively. For IFN-α stimulation experiments, 72 h after siRNA transfection, the cells were treated with 1,000 U/ml IFN-α for 30 h. For the TBK1 inhibitor experiments, the cells were pretreated with the BX795 inhibitor for 1 h and then treated with 1,000 U/ml IFN-α for 30 h.

### Plasmids, site-directed mutagenesis, viruses, and primers

For production of lentiviral particles, pLJM1-EGFP (19319), packaging vector psPAX2 (12260), and envelope vector pMD2.G (12259) were purchased from Addgene. For the construction of the pLJM1 plasmid encoding ADAR1p150, full-length human ADAR1p150 cDNA was amplified by PCR using the primers containing NheI and EcoRI restriction sites and inserted into pLJM1-EGFP. A mutation within ADARp150 (E912A) was introduced by site-directed mutagenesis as described previously [49]. The MISSION lentiviral shRNA plasmids encoding nontarget shRNA (SHC002), YTHDF1 shRNA (TRCN0000286871), and MDA5 shRNA (TRCN0000050849) were purchased from SIGMA. Lentiviruses were generated by transfection of 293T cells with lentiviral vectors, psPAX2 and pMD2.G. Lentiviral supernatants were collected at 48 and 72 h posttransfection, filtered, and added to A172 cells for 12 h. Stable transformants were selected with 1 μg/ml of puromycin and confirmed by immunoblotting for ADAR1 or YTHDF1. rVSV-GFP was generated by reverse genetics technique from the parental strain of VSV Indiana serotype in Li’s lab. A172 cells in 24-well plates were transfected with YTHDF1 siRNA or control siRNA, and 3 d later, the cells were infected with rVSV-GFP with the multiplicity of infection (MOI) of 0.1 at 37°C for 1 h. The virus inoculum was then removed, and 500 μL of DMEM supplemented with 2% FBS was added in each well. At the indicated time points postinfection, cells were photographed under the microscope, culture medium was collected for virus titration by plaque assay, and cells were lysed with RIPA buffer (Abcam, ab156034) for western blot sampling or TRizol reagent (Life Technologies, Carlsbad, California) for total RNA extraction.

### VSV plaque assay

Vero CCL81 cells were seeded in 6-well plates, and, at the next day, the confluent monolayer cells were inoculated with 0.5 ml 10-fold serial dilutions of collected samples in FBS-free DMEM at 37°C for 1 h. The inoculum was then replaced with 2 ml of DMEM overlay containing low-melting agarose (0.25% w/v), 5% FBS, 0.12% sodium bicarbonate, 100 μg/ml of streptomycin, 100 U/ml penicillin, 25 mM HEPES (pH 7.7), and 2 mM L-glutamine. After incubation at 37°C for 2 d, cells were fixed with 4% neutral buffered formaldehyde for 2 h. The overlay was then removed, and the cells were stained with 0.05% crystal violet to visualize the plaques. Virus titer was calculated with the formula of: Titer (Log_10_ PFU/ml) = Log_10_ (Plaque number / 0.5 × dilution).

### IFN-β ELISA

Spontaneous IFN-β production was detected by the VeriKine-HS Human IFN Beta Serum ELISA Kit (PBL Assay Science, 41515–1) according to the manufacturer’s instructions. A172 cells were seeded at a density of 70,000 cells per well in a 24-well plate. Next day, the cells were transfected with siRNAs by using the Lipofectamine RNAiMAX (Invitrogen). The cells were treated with 1,000 U/ml human IFN-α, 48 h after siRNA transfection. The cells were gently washed once with medium, then 350 μl fresh warm medium was replaced in the wells, 30 h after IFN-α stimulation. After 72 h of incubation, 50 μl of the supernatant was collected and the concentration of IFN-β was measured by the kit.

### RNA immunoprecipitation

The RNA immunoprecipitation (RIP) assay was performed as described previously [61] with minor modifications. Briefly, protein A Dynabeads (Invitrogen, 40 μl per 5 μg antibody) was washed with PBST buffer (PBS containing 0.05% Tween 20) 3 times and resuspended in 40 μl PBST buffer. The beads were incubated with 5 μg of control rabbit IgG (R&D SYSTEMS, AB105C), YTHDF1 (ProteinTech, 17479-1-AP) or YTHDF2 (ProteinTech, 24744-1-AP) antibody in 150 μl PBST buffer at 4°C overnight. A172 cells were collected by gentle scraping, pelleted by centrifuge for 5 min at 1,000*g*. The cell pellet was resuspended with lysis buffer (150 mM KCl, 10 mM Tris-HCl (pH 7.5), 2 mM EDTA, 0.5% NP-40, 0.5 mM DTT, 1% protease inhibitor cocktail, 400 U/ml RNase inhibitor), pipetted up and down several times. The mRNP lysate was incubated with rotation for 1 h at 4°C and centrifuged at 15,000*g* for 15 min to clear the lysate. An aliquot from each lysate was saved as input, mixed with TRIzol LS Reagent (Invitrogen). The cell lysate was mixed with antibody-coupled beads and rotated continuously for 3 h at 4°C. The beads were washed with 500 μl NT2 buffer (200 mM NaCl, 50 mM Tris-HCl (pH 7.5), 2 mM EDTA, 0.05% NP-40, 0.5 mM DTT, 0.1% protease inhibitor cocktail, 40 U/ml RNase inhibitor) 5 times and then resuspended in 90 μl ice-cold NT2 buffer. After the final wash, 10 μl of the beads were used for western blotting, and the remaining 80 μl were digested by proteinase K. The supernatant was mixed with TRIzol LS Reagent and saved as the IP sample. Total RNA isolated by TRIzol LS reagent was analyzed by RT-qPCR.

### Quantification of m^6^A level using anti-m^6^A antibody

The m^6^A-IP enrichment assay followed by RT-qPCR was performed as described previously [61]. Briefly, 3 μg purified cytosol mRNA extracted from A172 cells was incubated with 5 μg of control rabbit IgG (R&D SYSTEMS, AB105C) or m^6^A antibody (Synaptic Systems, 202003) for 4 h at 4°C, then pulled down by protein A Dynabeads (Invitrogen, 10008D) for 2 h at 4°C. RNA was extracted from the bead fractions by TRIzol LS Reagent, then subjected to RT-qPCR. HPRT1 gene was used as a reference gene when carrying out RT-qPCR.

### RNA preparation and RNA-seq

For quantitative RT-PCR and direct sequencing, total RNA was isolated by using RNeasy Mini Kit (QIAGEN) from cell lysate according to the manufacturer’s protocol. For quantitative RT-PCR in the samples of VSV-infected cells, total RNA was extracted from TRizol-lysate and dissolved in 50 μl water. For RNA-seq experiments, total RNA was isolated by using TRIzol reagent (Invitrogen) from cell lysate, and poly (A)+ RNA was purified from the total RNA by using Dynabeads mRNA DIRECT Purification Kit (Invitrogen). The RNA libraries were prepared using Truseq stranded mRNA sample preparation kit (Illumina) according to the manufacturer’s protocol. Three biological replicates were sequenced on an Illumina HiSeq 4000 sequencer (50 bp, single end) for each cell lines.

### Quantitative RT-PCR (RT-qPCR)

To quantify expression levels of transcripts, total RNA was reverse transcribed by Go Script Reverse Transcriptase (Promega, A6010) with anchored (dT)15 primers and random oligo primers. The cDNA was subjected to real-time PCR (StepOnePlus Real-Time PCR Systems: Applied Biosystems) by using GoTaq Master Mix (Promega, A6010) with the gene specific primers (S1 Table). Relative changes in expression were calculated using the ΔΔCt method. To quantify expression levels of VSV genomic RNA, 2 μl of total RNA was used for cDNA synthesis in 20 μl RT system with primer annealed to 3′-end leader sequence of VSV genome (VSV genome-RT). To quantify expression levels of VSV antigenomic RNA, 2 μl of total RNA was used for cDNA synthesis with primer annealed to 5′-end trailer sequence of VSV genome (VSV antigenome-RT). The same pair of primers annealing to G gene were used for RT-qPCR for VSV genome and antigenome with 2 μl of cDNA. The Ct values were converted into genomic RNA or total viral RNA copies according to the standard curve generated with serial dilutions of VSV full-length genome plasmid. *GAPDH* (for mRNA expression level) and *HPRT1* (for m^6^A-IP) were used as internal controls.

### Direct sequencing

A-to-I RNA editing level was measured as described previously [31]. Briefly, total RNA was reverse transcribed by Go Script Reverse Transcriptase with anchored (dT)15 primers and random oligo primers. The cDNAs were amplified by Phusion Hot Start II High-Fidelity DNA Polymerase (Thermo Scientific, F549) with gene specific primers (S1 Table, Fw1 and Rv1). Second PCR reactions were performed with the second sets of gene specific primers (S1 Table, Fw2 and Rv2) and with the first RT-PCR products (diluted 80-fold) as templates. Subsequently, the amplified cDNA fragments from RT-PCR were treated with ExoSAP-IT (USB) and directly sequenced with the gene specific primer, Fw2 or Rv2. The editing levels were quantified by measuring heights of A peaks (unedited) and G peaks (edited) and calculating percentage of the population edited at each site (100% × [G height / (A height + G height)]).

### Measuring decay rates of mRNAs

Cells were transfected with control siRNA and YTHDF1 siRNA by using the Lipofectamine RNAiMAX. The cells were treated with 1,000 U/ml IFN-α for 30 h, 72 h after siRNA transfection. The cells were treated with 1 μg/ml Actinomycin D for time periods specified in S2A Fig. Total RNA was isolated from the cells by using RNeasy Mini Kit and analyzed by RT-qPCR.

### Cell proliferation and apoptosis assay

For cell proliferation assay, 2,500 cells of the stable cell lines expressing control shRNA or YTHDF1 shRNA were seeded per well in a 96-well plate with mock treatment or IFN-α stimulation. The cell proliferation was measured by using Cell Counting Kit-8 (Dojindo Molecular Technologies, CK04) at various time points according to the manufacturer’s protocols. For each cell line tested, the signal from the assay was normalized to the value obtained from 24 h after seeding.

For apoptosis assay, 30,000 cells of the stable cell lines expressing control shRNA or YTHDF1 shRNA were seeded per well in a 24-well plate with mock treatment or IFN-α stimulation. After 5 d of incubation with IFN-α, apoptotic cells were stained with a MEBSTAIN Apoptosis TUNEL Kit Direct (MBL International, 8445) according to the manufacturer’s protocol, and the stained cells were examined by flow cytometry.

### RNA-seq data processing

Sequenced reads were mapped to the human genome by HiSAT2 [62] (version 2.1.0) with parameters “—score-min L,0,-0.6.” For estimation of gene expression levels, the mapped reads of each sample were assembled in a reference-based approach, and transcripts per million (TPM) was calculated for each genes by using StringTie [63] (version 2.0). Differentially expressed genes were detected by R package DESeq2 [64] (version 1.24.0) using Wald test. The significantly differentially expressed genes between control siRNA and YTHDF1 knockdown were reported at adjusted *p-*value cutoff of 0.05. Heatmaps were generated by heatmap2 in gplots package of R.

### Quantification of A-to-I RNA editing levels

Sequenced reads were mapped to the human genome by HiSAT2 [62] (version 2.1.0) with parameters “—score-min L,0,-0.6.” Known A-to-I RNA editing sites in the human transcripts were obtained from 2 databases, DARNED and REDIportal [45,46]. A-to-I RNA editing levels for the list of known editing sites were calculated by using REDItools [65] algorithm REDItoolKnown.py with parameters “-v 0 -n 0 -m 60.” Among 4,688,186 editing sites, 6,588 sites fulfilled our first criteria (termed “Depth filter”): minimum depth of coverage >10× (more than 10 reads at every samples in our RNA-seq data). Among the 6,588 sites, 488 sites fulfilled our second criteria (“Editing filter”): maximum average editing level >5%. We also excluded the editing sites exhibiting minimum average editing level <95%, because it could be caused by genomic mutation in this cell line. An average editing level was defined as “increased/decreased” if a difference between control and IFN-α stimulation/YTHDF1 knockdown was more than 5%. Heatmaps were generated by heatmap2 in gplots package of R.

### Quantification and statistical analysis

Two-tailed Student *t* tests were performed to assess the statistical significance of differences between groups, *p < 0.05, **p < 0.01, ***p < 0.001, n.s. *p* ≧ 0.05. N.D. means not detected. *n =* 3 for all experiments. Data are presented as the mean ± standard error of the mean (SEM).

### Reference sequence and annotation data

The human genome sequence (GRCh38/hg38), the annotated gene models, and the annotations of rRNAs were obtained from the UCSC genome browser database.

## Supporting information

S1 Fig m^6^A effects on *ADAR1* and *ADAR2* mRNAs. (A) m^6^A-seq data of the *ADAR3* transcript in HepG2 cells, modified from Dominissini and colleagues [9]. An arrow indicates the transcription direction. (B, C) Immunoblot analysis showing knockdown effects of METTL3, METTL14, YTHDF1, and YTHDF2 on ADAR1p150, ADAR1p110, and ADAR2 protein expression levels in A172 cells under normal culture conditions. Immunoblot images are representative of 3 biological replicates. (D) RT-qPCR showing knockdown effect of YTHDF2 on *ADAR2* mRNA. The signals were normalized to *GAPDH*. (E) Immunoblot analysis showing knockdown effects of METTL3, METTL14, YTHDF1, and YTHDF2 on ADAR1p150 and ADAR1p110 protein expression levels following IFN-α stimulation. Immunoblot images are representative of 3 biological replicates. (F) RT-qPCR showing knockdown effects of METTL3 and YTHDF1 on *ADAR1p150* mRNA following IFN-α stimulation. The signals were normalized to *GAPDH*. (G) Immunoblot analysis showing time course of ADAR1p150 protein degradation in A172 cells after IFN-α stimulation. Cells were collected at 0, 12, and 24 h after the addition of CHX. ADAR1p150 protein levels at the starting point were normalized to 1. (H) Effects of METTL3 and YTHDF1 knockdown on *ADAR1p150* translation efficiency in A172 cells following IFN-α stimulation (ratio of protein amounts to mRNA levels). (I) Quantification of ADAR1p150 protein expression levels from immunoblot images of Fig 1E. (C, D, F–I) Two-tailed Student *t* tests were performed to assess the statistical significance of differences between groups, *p < 0.05, **p < 0.01, ***p < 0.001. *n =* 3 for all experiments. Data are presented as the mean ± SEM. The numerical values for this figure are available in S1 Data. CHX, cycloheximide; IP, immunoprecipitation; m^6^A, *N*^6^-methyladenosine; n.s., not significant; RT-qPCR, quantitative reverse transcription PCR; SEM, standard error of the mean; siRNA, small interfering RNA.
(TIF)

S2 Fig m^6^A effects on IFN genes and ISGs. (A) Decay profiles of *IFNB1* and *IFNL1* mRNAs in control or YTHDF1 knockdown cells that were pretreated with IFN-α and then treated with actinomycin D. The signals were normalized to signals at the time 0 h. (B) Quantification of protein expression levels from immunoblot images of Fig 3C and 3H. (C) A-to-I RNA editing of a few selected transcripts in stable cell lines expressing control EGFP, wild-type ADAR1p150, or catalytically inactive mutant of ADAR1p150^E912A^, respectively. All cells were transfected with YTHDF1 siRNA and treated with IFN-α. (D) RT-qPCR showing inhibitory effects of BX795 on IFNs mRNA induction upon poly (I:C) stimulation. A172 cells that were pretreated with the BX759 inhibitor for 1 h and then transfected with poly (I:C). (E) RT-qPCR showing that knockdown efficiency of MDA5 in the samples of Fig 4D. (D, E) The signals were normalized to *GAPDH*. (A–E) Two-tailed Student *t* tests were performed to assess the statistical significance of differences between groups, *p < 0.05, **p < 0.01, ***p < 0.001, n.s. *p* ≧ 0.05, N.D. means not detected. *n =* 3 for all experiments. Data are presented as the mean ± SEM. The numerical values for this figure are available in S1 Data. A-to-I RNA editing, adenosine-to-inosine RNA editing; IFN, interferon; ISG, IFN-stimulated gene; m^6^A, *N*^6^-methyladenosine; N.D., not detected; n.s., not significant; RT-qPCR, quantitative reverse transcription PCR; SEM, standard error of the mean; siRNA, small interfering RNA; TBK1, TANK-binding kinase 1.
(TIF)

S3 Fig YTHDF1 affects cell proliferation and apoptosis upon IFN stimulation. (A) Immunoblot analyzing in stable YTHDF1 knockdown cells following IFN-α stimulation. Immunoblot images are representative of 3 biological replicates. (B) RT-qPCR showing transcripts levels of *IFNB1*, *IFNL1*, and *IFNL3* in stable YTHDF1 knockdown cells versus controls following IFN-α stimulation. The signals were normalized to *GAPDH*. (C) IFN-responsive cell proliferation rate in stable YTHDF1 knockdown and ADAR1p150 overexpressing cell lines. (D) RT-qPCR of *MDA5* mRNAs in stable MDA5 knockdown A172 cells. The signals were normalized to *GAPDH*. (E) IFN-responsive cell proliferation rate in stable YTHDF1 and MDA5 knockdown cell lines. (F) IFN-induced apoptosis measured by TUNEL assay in stable YTHDF1 knockdown cells following IFN-α stimulation. YTHDF1 knockdown increases apoptosis. (G) IFN-induced apoptosis measured by TUNEL assay in stable YTHDF1 and MDA5 knockdown cells following IFN-α stimulation. (B–G) Two-tailed Student *t* tests were performed to assess the statistical significance of differences between groups, *p < 0.05, **p < 0.01, ***p < 0.001, n.s. *p* ≧ 0.05. *n =* 3 for all experiments. Data are presented as the mean ± SEM. The numerical values for this figure are available in S1 Data. IFN, interferon; n.s., not significant; RT-qPCR, quantitative reverse transcription PCR; SEM, standard error of the mean; shRNA, short hairpin RNA.
(TIF)

S4 Fig YTHDF1 affects cell proliferation and apoptosis upon IFN stimulation. (A) Immunoblot analysis showing knockdown effects of YTHDF1 on ADAR1p150 protein expression following IFN-α stimulation in LN229, HeLa, and HEK293T cells. Immunoblot images are representative of 3 biological replicates. (B) RT-qPCR showing knockdown effects of YTHDF1 on *ADAR1p150* mRNA following IFN-α stimulation in LN229, HeLa, and HEK293T cells. (C) RT-qPCR showing knockdown effect of YTHDF1 on IFN genes, ISGs, and NF-κB–inducible genes in LN229 cells. (D) RT-qPCR showing knockdown effect on *YTHDF1* mRNA in stable YTHDF1 knockdown LN229 cells. (E) IFN-responsive cell proliferation rate in stable YTHDF1 knockdown LN229 cells. (F) IFN-induced apoptosis measured by TUNEL assay in stable YTHDF1 knockdown LN229 cells following IFN-α stimulation. (B–D) The signals were normalized to *GAPDH*. (B–F) Two-tailed Student *t* tests were performed to assess the statistical significance of differences between groups, *p < 0.05, **p < 0.01, ***p < 0.001, n.s. *p* ≧ 0.05, N.D. means not detected. *n =* 3 for all experiments. Data are presented as the mean ± SEM. The numerical values for this figure are available in S1 Data. IFN, interferon; ISG, IFN-stimulated gene; n.s., not significant; RT-qPCR, quantitative reverse transcription PCR; SEM, standard error of the mean; shRNA, short hairpin RNA.
(TIF)

S5 Fig YTHDF1 knockdown inhibits viral replication. (A) YTHDF1 knockdown decreases GFP expression in rVSV-infected A172 cells. A172 cells transfected with YTHDF1 siRNA or control siRNA were infected with rVSV-GFP at an MOI of 0.1, and GFP expression was monitored at 12, 20, and 24 h postinfection. Cells are shown in the same area under bright-field and fluorescence images. (B, C) Immunoblot analysis showing significant decrease in the expression of VSV-G protein upon YTHDF1 knockdown at 12 and 20 h after rVSV-GFP infection. Immunoblot images are representative of 3 biological replicates. (D, E) Immunoblot analysis showing knockdown effect of YTHDF1 on the expression of VSV-G protein at 12 and 20 h after rVSV-GFP infection in stable cell lines expressing control EGFP, wild-type ADAR1p150, or catalytically inactive mutant of ADAR1p150^E912A^, respectively. Immunoblot images are representative of 3 biological replicates. The signals were normalized to control siRNA samples. (C, E) Two-tailed Student *t* tests were performed to assess the statistical significance of differences between groups, *p < 0.05, **p < 0.01, ***p < 0.001, n.s. *p* ≧ 0.05. *n =* 3 for all experiments. Data are presented as the mean ± SEM. The numerical values for this figure are available in S1 Data. MOI, multiplicity of infection; n.s., not significant; rVSV-GFP, recombinant GFP-expressing vesicular stomatitis virus; SEM, standard error of the mean; siRNA, small interfering RNA.
(TIF)

S6 Fig A-to-I RNA editing sites in several IFN-induced transcripts. (A) RT-qPCR of *EIF2AK2*, *TNKS*, *PSMB2*, *RSAD2*, *STAT1*, *CFLAR*, and *ZC3HAV1* mRNAs in A172 cells. The signals were normalized to GAPDH. (B) Expression levels of *TRIM56*, *IFI44L*, *CD74*, and *TAP1* mRNAs in RNA-seq analysis of A172 cells. (C) Direct sequencing chromatogram showing from RT-PCR products of *RSAD2*, *STAT1*, *TRIM56*, *IFI44L*, *CD74*, *TAP1*, *CFLAR*, and *ZC3HAV1* mRNA following IFN-α stimulation. (D) A172 cells were transfected with YTHDF1 siRNA or control siRNA and treated with IFN-α. The cells were then stained with J2 antibody and fluorescent secondary antibody and analyzed by flow cytometry. (E) A schematic model showing YTHDF1-mediated regulation of IFN responses. (A, B, D) Two-tailed Student *t* tests were performed to assess the statistical significance of differences between groups, *p < 0.05, **p < 0.01, ***p < 0.001. *n* = 3 for all experiments. Data are presented as the mean ± SEM. The numerical values for this figure are available in S1 Data. A-to-I RNA editing, adenosine-to-inosine RNA editing; dsRNA, double-stranded RNA; IFN, interferon; IRF3, IFN regulatory factor 3; m^6^A, *N*^6^-methyladenosine; RT-qPCR, quantitative reverse transcription PCR; SEM, standard error of the mean; siRNA, small interfering RNA; TBK1, TANK-binding kinase 1; TPM, transcripts per kilobase million.
(TIF)

S1 Table RT-qPCR primers, siRNAs, antibodies, and direct-sequencing primers. (XLSX)

S2 Table A-to-I RNA editing levels in RNA-seq analysis. (XLSX)

S3 Table Gene expression profiles in RNA-seq analysis. (XLSX)

S1 Data Excel spreadsheet containing the underlying numerical values for figure panels Figs 1B, 1D, 2C, 2D, 3A, 3B, 3D–3G, 4B–4D, 5A–5F, S1C, S1D, S1F–S1I, S2A–S2E, S3B–S3G, S4A–S4F, S5C, S5E, S6A, S6B and S6D. (XLSX)

S1 Raw Images Original images supporting all immunoblot analysis reported in Figs 1C, 1E, 3C, 3H, 4A, S1B, S1E, S3A, S4A, S5B and S5D. (PDF)

## Abbreviations

ADAR: adenosine deaminase acting on RNA
AGS: Aicardi–Goutières syndrome
ALKBH5: alkB homolog 5, RNA demethylase
A-to-I RNA editing: adenosine-to-inosine RNA editing
CHX: cycloheximide
DMEM: Dulbecco’s modified Eagle’s medium
dsRNA: double-stranded RNA
FBS: fetal bovine serum
FTO: Fat mass and obesity-associated gene
HCMV: human cytomegalovirus
IFIH1: interferon induced with helicase C domain 1
IFN: interferon
IFNB: interferon beta
IFNL: interferon lambda
IRF3: IFN regulatory factor 3
ISG: IFN-stimulated gene
MAVS: mitochondrial antiviral-signaling protein
METTL: methyltransferase-like
MOI: multiplicity of infection
m^6^A: *N*^6^-methyladenosine
m^6^A-IP: m^6^A immunoprecipitation
RIG-I: retinoic acid–inducible gene I
RIP: RNA immunoprecipitation
RLR: RIG-I–like receptor
RT: reverse transcription
RT-qPCR: quantitative reverse transcription PCR
rVSV-GFP: recombinant GFP-expressing vesicular stomatitis virus
shRNA: short hairpin RNA
TBK1: TANK-binding kinase 1
TPM: transcripts per million
YTHDF: YTH *N*^6^-methyladenosine RNA binding protein

## References

[1] Boccaletto P, Machnicka MA, Purta E, Piatkowski P, Baginski B, Wirecki TK, et al. MODOMICS: a database of RNA modification pathways. 2017 update. Nucleic Acids Res. 2018;46:D303–7. doi: 10.1093/nar/gkx1030 29106616 PMC5753262

[2] Helm M, Motorin Y. Detecting RNA modifications in the epitranscriptome: predict and validate. Nat Rev Genet. 2017;18:275–91. doi: 10.1038/nrg.2016.169 28216634

[3] Roundtree IA, Evans ME, Pan T, He C. Dynamic RNA Modifications in Gene Expression Regulation. Cell. 2017;169:1187–200. doi: 10.1016/j.cell.2017.05.045 28622506 PMC5657247

[4] Zhao BS, Roundtree IA, He C. Post-transcriptional gene regulation by mRNA modifications. Nat Rev Mol Cell Biol. 2017;18:31–42. doi: 10.1038/nrm.2016.132 27808276 PMC5167638

[5] Liu J, Harada BT, He C. Regulation of Gene Expression by N6-methyladenosine in Cancer. Trends Cell Biol. 2019;29:487–99. doi: 10.1016/j.tcb.2019.02.008 30940398 PMC6527461

[6] Bokar JA, Shambaugh ME, Polayes D, Matera AG, Rottman FM. Purification and cDNA cloning of the AdoMet-binding subunit of the human mRNA (N6-adenosine)-methyltransferase. RNA. 1997;3:1233–47. 9409616 PMC1369564

[7] Liu J, Yue Y, Han D, Wang X, Fu Y, Zhang L, et al. A METTL3-METTL14 complex mediates mammalian nuclear RNA N6-adenosine methylation. Nat Chem Biol. 2014;10:93–5. doi: 10.1038/nchembio.1432 24316715 PMC3911877

[8] Wang Y, Li Y, Toth JI, Petroski MD, Zhang Z, Zhao JC. N6-methyladenosine modification destabilizes developmental regulators in embryonic stem cells. Nat Cell Biol. 2014;16:191–8. doi: 10.1038/ncb2902 24394384 PMC4640932

[9] Dominissini D, Moshitch-Moshkovitz S, Schwartz S, Salmon-Divon M, Ungar L, Osenberg S, et al. Topology of the human and mouse m6A RNA methylomes revealed by m6A-seq. Nature. 2012;485:201–6. doi: 10.1038/nature11112 22575960

[10] Wang X, Zhao BS, Roundtree IA, Lu Z, Han D, Ma H, et al. N6-methyladenosine Modulates Messenger RNA Translation Efficiency. Cell. 2015;161:1388–99. doi: 10.1016/j.cell.2015.05.014 26046440 PMC4825696

[11] Zhang Z, Luo K, Zou Z, Qiu M, Tian J, Sieh L, et al. Genetic analyses support the contribution of mRNA N 6 -methyladenosine (m 6 A) modification to human disease heritability. Nat Genet. 2020:1–11. doi: 10.1038/s41588-020-0644-z 32601472 PMC7483307

[12] Wang X, Lu Z, Gomez A, Hon GC, Yue Y, Han D, et al. N6-methyladenosine-dependent regulation of messenger RNA stability. Nature. 2014;505:117–120. doi: 10.1038/nature12730 24284625 PMC3877715

[13] Du H, Zhao Y, He J, Zhang Y, Xi H, Liu M, et al. YTHDF2 destabilizes m(6)A-containing RNA through direct recruitment of the CCR4-NOT deadenylase complex. Nat Commun. 2016;7:12626. doi: 10.1038/ncomms12626 27558897 PMC5007331

[14] Jia G, Fu Y, Zhao X, Dai Q, Zheng G, Yang Y, et al. N6-methyladenosine in nuclear RNA is a major substrate of the obesity-associated FTO. Nat Chem Biol. 2011;7:885–7. doi: 10.1038/nchembio.687 22002720 PMC3218240

[15] Zheng G, Dahl JA, Niu Y, Fedorcsak P, Huang C-M, Li CJ, et al. ALKBH5 is a mammalian RNA demethylase that impacts RNA metabolism and mouse fertility. Mol Cell. 2013;49:18–29. doi: 10.1016/j.molcel.2012.10.015 23177736 PMC3646334

[16] He C. Grand challenge commentary: RNA epigenetics? Nat Chem Biol. 2010;6:863–5. doi: 10.1038/nchembio.482 21079590

[17] Hogg M, Paro S, Keegan LP. O’Connell MA. RNA editing by mammalian ADARs. Adv Genet. 2011;73:87–120. doi: 10.1016/B978-0-12-380860-8.00003-3 21310295

[18] Eisenberg E. Levanon EY. A-to-I RNA editing—immune protector and transcriptome diversifier. Nat Rev Genet. 2018;19:473–90. doi: 10.1038/s41576-018-0006-1 29692414

[19] Nishikura K. A-to-I editing of coding and non-coding RNAs by ADARs. Nat Rev Mol Cell Biol. 2016;17:83–96. doi: 10.1038/nrm.2015.4 26648264 PMC4824625

[20] Solomon O, Oren S, Safran M, Deshet-Unger N, Akiva P, Jacob-Hirsch J, et al. Global regulation of alternative splicing by adenosine deaminase acting on RNA (ADAR). RNA. 2013;19:591–604. doi: 10.1261/rna.038042.112 23474544 PMC3677275

[21] Porath HT, Knisbacher BA, Eisenberg E, Levanon EY. Massive A-to-I RNA editing is common across the Metazoa and correlates with dsRNA abundance. Genome Biol. 2017;18:185. doi: 10.1186/s13059-017-1315-y 28969707 PMC5625713

[22] Solomon O, Di Segni A, Cesarkas K, Porath HT, Marcu-Malina V, Mizrahi O, et al. RNA editing by ADAR1 leads to context-dependent transcriptome-wide changes in RNA secondary structure. Nat Commun. 2017;8:1440. doi: 10.1038/s41467-017-01458-8 29129909 PMC5682290

[23] Samuel CE. Adenosine deaminase acting on RNA (ADAR1), a suppressor of double-stranded RNA–triggered innate immune responses. J Biol Chem. 2019;294:1710–20. doi: 10.1074/jbc.TM118.004166 30710018 PMC6364763

[24] Hartner JC, Walkley CR, Lu J, Orkin SH. ADAR1 is essential for the maintenance of hematopoiesis and suppression of interferon signaling. Nat Immunol. 2009;10:109–15. doi: 10.1038/ni.1680 19060901 PMC2701568

[25] Liddicoat BJ, Piskol R, Chalk AM, Ramaswami G, Higuchi M, Hartner JC, et al. RNA editing by ADAR1 prevents MDA5 sensing of endogenous dsRNA as nonself. Science. 2015. doi: 10.1126/science.aac7049 26275108 PMC5444807

[26] Pestal K, Funk CC, Snyder JM, Price ND, Treuting PM, Stetson DB. Isoforms of RNA-Editing Enzyme ADAR1 Independently Control Nucleic Acid Sensor MDA5-Driven Autoimmunity and Multi-organ Development. Immunity. 2015;43:933–44. doi: 10.1016/j.immuni.2015.11.001 26588779 PMC4654992

[27] Rice GI, Kasher PR, Forte GMA, Mannion NM, Greenwood SM, Szynkiewicz M, et al. Mutations in ADAR1 cause Aicardi-Goutières syndrome associated with a type I interferon signature. Nat Genet. 2012. doi: 10.1038/ng.2414 23001123 PMC4154508

[28] Behm M, Öhman M. RNA Editing: A Contributor to Neuronal Dynamics in the Mammalian Brain. Trends Genet. 2016. doi: 10.1016/j.tig.2015.12.005 26803450

[29] Tariq A, Jantsch MF. Transcript Diversification in the Nervous System: A to I RNA Editing in CNS Function and Disease Development. Front Neurosci. 2012;6:99. doi: 10.3389/fnins.2012.00099 22787438 PMC3391646

[30] Terajima H, Yoshitane H, Ozaki H, Suzuki Y, Shimba S, Kuroda S, et al. ADARB1 catalyzes circadian A-to-I editing and regulates RNA rhythm. Nat Genet. 2017;49:146–51. doi: 10.1038/ng.3731 27893733

[31] Terajima H, Yoshitane H, Yoshikawa T, Shigeyoshi Y, Fukada Y. A-to-I RNA editing enzyme ADAR2 regulates light-induced circadian phase-shift. Sci Rep. 2018;8:14848. doi: 10.1038/s41598-018-33114-6 30287844 PMC6172258

[32] Kung C-P, Maggi LB, Weber JD. The Role of RNA Editing in Cancer Development and Metabolic Disorders. Front Endocrinol. 2018;9:762. doi: 10.3389/fendo.2018.00762 30619092 PMC6305585

[33] Liu H, Golji J, Brodeur LK, Chung FS, Chen JT, deBeaumont RS, et al. Tumor-derived IFN triggers chronic pathway agonism and sensitivity to ADAR loss. Nat Med. 2019;25:95–102. doi: 10.1038/s41591-018-0302-5 30559422

[34] Gannon HS, Zou T, Kiessling MK, Gao GF, Cai D, Choi PS, et al. Identification of ADAR1 adenosine deaminase dependency in a subset of cancer cells. Nat Commun. 2018;9:5450. doi: 10.1038/s41467-018-07824-4 30575730 PMC6303303

[35] Ishizuka JJ, Manguso RT, Cheruiyot CK, Bi K, Panda A, Iracheta-Vellve A, et al. Loss of ADAR1 in tumours overcomes resistance to immune checkpoint blockade. Nature. 2018;1. doi: 10.1038/s41586-018-0768-9 30559380 PMC7241251

[36] Ma L, Zhao B, Chen K, Thomas A, Tuteja JH, He X, et al. Evolution of transcript modification by N(6)-methyladenosine in primates. Genome Res. 2017;27:385–92. doi: 10.1101/gr.212563.116 28052920 PMC5340966

[37] Xiang J-F, Yang Q, Liu C-X, Wu M, Chen L-L, Yang L. N6-Methyladenosines Modulate A-to-I RNA Editing. Mol Cell. 2018;69:126–135.e6. doi: 10.1016/j.molcel.2017.12.006 29304330

[38] Meyer KD, Saletore Y, Zumbo P, Elemento O, Mason CE, Jaffrey SR. Comprehensive analysis of mRNA methylation reveals enrichment in 3’ UTRs and near stop codons. Cell. 2012;149:1635–46. doi: 10.1016/j.cell.2012.05.003 22608085 PMC3383396

[39] Zhou J, Wan J, Gao X, Zhang X, Jaffrey SR, Qian S-B. Dynamic m6A mRNA methylation directs translational control of heat shock response. Nature. 2015;526:591–4. doi: 10.1038/nature15377 26458103 PMC4851248

[40] Meyer KD, Patil DP, Zhou J, Zinoviev A, Skabkin MA, Elemento O, et al. 5’ UTR m6A Promotes Cap-Independent Translation. Cell. 2015;163:999–1010. doi: 10.1016/j.cell.2015.10.012 26593424 PMC4695625

[41] Anders M, Chelysheva I, Goebel I, Trenkner T, Zhou J, Mao Y, et al. Dynamic m6A methylation facilitates mRNA triaging to stress granules. Life Sci Alliance. 2018;e201800113:1. doi: 10.26508/lsa.201800113 30456371 PMC6238392

[42] Patterson JB, Samuel CE. Expression and regulation by interferon of a double-stranded-RNA-specific adenosine deaminase from human cells: evidence for two forms of the deaminase. Mol Cell Biol. 1995;15:5376–88. doi: 10.1128/MCB.15.10.5376 7565688 PMC230787

[43] Shi H, Zhang X, Weng Y-L, Lu Z, Liu Y, Lu Z, et al. m6A facilitates hippocampus-dependent learning and memory through YTHDF1. Nature. 2018;563:249–53. doi: 10.1038/s41586-018-0666-1 30401835 PMC6226095

[44] McFadden MJ, McIntyre ABR, Mourelatos H, Abell NS, Gokhale NS, Ipas H, et al. Post-transcriptional regulation of antiviral gene expression by N6-methyladenosine. Cell Rep. 2021:34. doi: 10.1016/j.celrep.2021.108798 33657363 PMC7981787

[45] Kiran AM, O’Mahony JJ, Sanjeev K, Baranov PV. Darned in 2013: inclusion of model organisms and linking with Wikipedia. Nucleic Acids Res. 2013;41:D258–61. doi: 10.1093/nar/gks961 23074185 PMC3531090

[46] Picardi E, D’Erchia AM, Lo Giudice C, Pesole G. REDIportal: a comprehensive database of A-to-I RNA editing events in humans. Nucleic Acids Res. 2017;45:D750–7. doi: 10.1093/nar/gkw767 27587585 PMC5210607

[47] Rubio RM, Depledge DP, Bianco C, Thompson L. Mohr I. RNA m6 A modification enzymes shape innate responses to DNA by regulating interferon β. Genes Dev. 2018;32:1472–84. doi: 10.1101/gad.319475.118 30463905 PMC6295168

[48] Winkler R, Gillis E, Lasman L, Safra M, Geula S, Soyris C, et al. m6A modification controls the innate immune response to infection by targeting type I interferons. Nat Immunol. 2019;20:173–82. doi: 10.1038/s41590-018-0275-z 30559377

[49] Lai F, Drakas R, Nishikura K. Mutagenic analysis of double-stranded RNA adenosine deaminase, a candidate enzyme for RNA editing of glutamate-gated ion channel transcripts. J Biol Chem. 1995;270:17098–105. doi: 10.1074/jbc.270.29.17098 7615504

[50] Qiu W, Zhang Q, Zhang R, Lu Y, Wang X, Tian H, et al. N 6 -methyladenosine RNA modification suppresses antiviral innate sensing pathways via reshaping double-stranded RNA. Nat Commun. 1582;2021:12. doi: 10.1038/s41467-021-21904-y 33707441 PMC7952553

[51] Lu M, Zhang Z, Xue M, Zhao BS, Harder O, Li A, et al. N6-methyladenosine modification enables viral RNA to escape recognition by RNA sensor RIG-I. Nat Microbiol. 2020. doi: 10.1038/s41564-019-0653-9 32015498 PMC7137398

[52] Jin D, Guo J, Wu Y, Yang L, Wang X, Du J, et al. m6A demethylase ALKBH5 inhibits tumor growth and metastasis by reducing YTHDFs-mediated YAP expression and inhibiting miR-107/LATS2-mediated YAP activity in NSCLC. Mol Cancer. 2020;19:40. doi: 10.1186/s12943-020-01161-1 32106857 PMC7045432

[53] Han B, Yan S, Wei S, Xiang J, Liu K, Chen Z, et al. YTHDF1-mediated translation amplifies Wnt-driven intestinal stemness. EMBO Rep. 2020;21:e49229. doi: 10.15252/embr.201949229 32064749 PMC7132202

[54] Zhuang M, Li X, Zhu J, Zhang J, Niu F, Liang F, et al. The m6A reader YTHDF1 regulates axon guidance through translational control of Robo3.1 expression. Nucleic Acids Res. 2019;47:4765–77. doi: 10.1093/nar/gkz157 30843071 PMC6511866

[55] Han D, Liu J, Chen C, Dong L, Liu Y, Chang R, et al. Anti-tumour immunity controlled through mRNA m 6 A methylation and YTHDF1 in dendritic cells. Nature. 2019;566:270. doi: 10.1038/s41586-019-0916-x 30728504 PMC6522227

[56] Zaccara S, Jaffrey SR. A Unified Model for the Function of YTHDF Proteins in Regulating m6A-Modified mRNA. Cell. 2020 [cited 2020 Jun 6]. doi: 10.1016/j.cell.2020.05.012 32492408 PMC7508256

[57] Xue M, Zhao BS, Zhang Z, Lu M, Harder O, Chen P, et al. Viral N6-methyladenosine upregulates replication and pathogenesis of human respiratory syncytial virus. Nat Commun. 2019;10:1–18. doi: 10.1038/s41467-018-07882-8 31597913 PMC6785563

[58] Zheng Q, Hou J, Zhou Y, Li Z, Cao X. The RNA helicase DDX46 inhibits innate immunity by entrapping m6A-demethylated antiviral transcripts in the nucleus. Nat Immunol. 2017;18:1094–103. doi: 10.1038/ni.3830 28846086

[59] Porritt RA, Hertzog PJ. Dynamic control of type I IFN signalling by an integrated network of negative regulators. Trends Immunol. 2015;36:150–60. doi: 10.1016/j.it.2015.02.002 25725583

[60] Fritzell K, Xu L-D, Lagergren J, Öhman M. ADARs and editing: The role of A-to-I RNA modification in cancer progression. Semin Cell Dev Biol. 2017. doi: 10.1016/j.semcdb.2017.11.018 29146145

[61] Liu J, Eckert MA, Harada BT, Liu S-M, Lu Z, Yu K, et al. m6A mRNA methylation regulates AKT activity to promote the proliferation and tumorigenicity of endometrial cancer. Nat Cell Biol. 2018;20:1074–83. doi: 10.1038/s41556-018-0174-4 30154548 PMC6245953

[62] Kim D, Langmead B, Salzberg SL. HISAT: a fast spliced aligner with low memory requirements. Nat Methods. 2015;12:357–60. doi: 10.1038/nmeth.3317 25751142 PMC4655817

[63] Pertea M, Pertea GM, Antonescu CM, Chang T-C, Mendell JT, Salzberg SL. StringTie enables improved reconstruction of a transcriptome from RNA-seq reads. Nat Biotechnol. 2015;33:290–5. doi: 10.1038/nbt.3122 25690850 PMC4643835

[64] Love MI, Huber W, Anders S. Moderated estimation of fold change and dispersion for RNA-seq data with DESeq2. Genome Biol. 2014;15:550. doi: 10.1186/s13059-014-0550-8 25516281 PMC4302049

[65] Picardi E, Pesole G. REDItools: high-throughput RNA editing detection made easy. Bioinformatics. 2013;29:1813–4. doi: 10.1093/bioinformatics/btt287 23742983

