# Supplementary figures and images for: N6-methyladenosine promotes induction of ADAR1-mediated A-to-I RNA editing to suppress aberrant antiviral innate immune responses
Source: PLoS Biol. 2021 Jul 29;19(7):e3001292. doi: 10.1371/journal.pbio.3001292 (PMC8320976; doi:10.1371/journal.pbio.3001292)

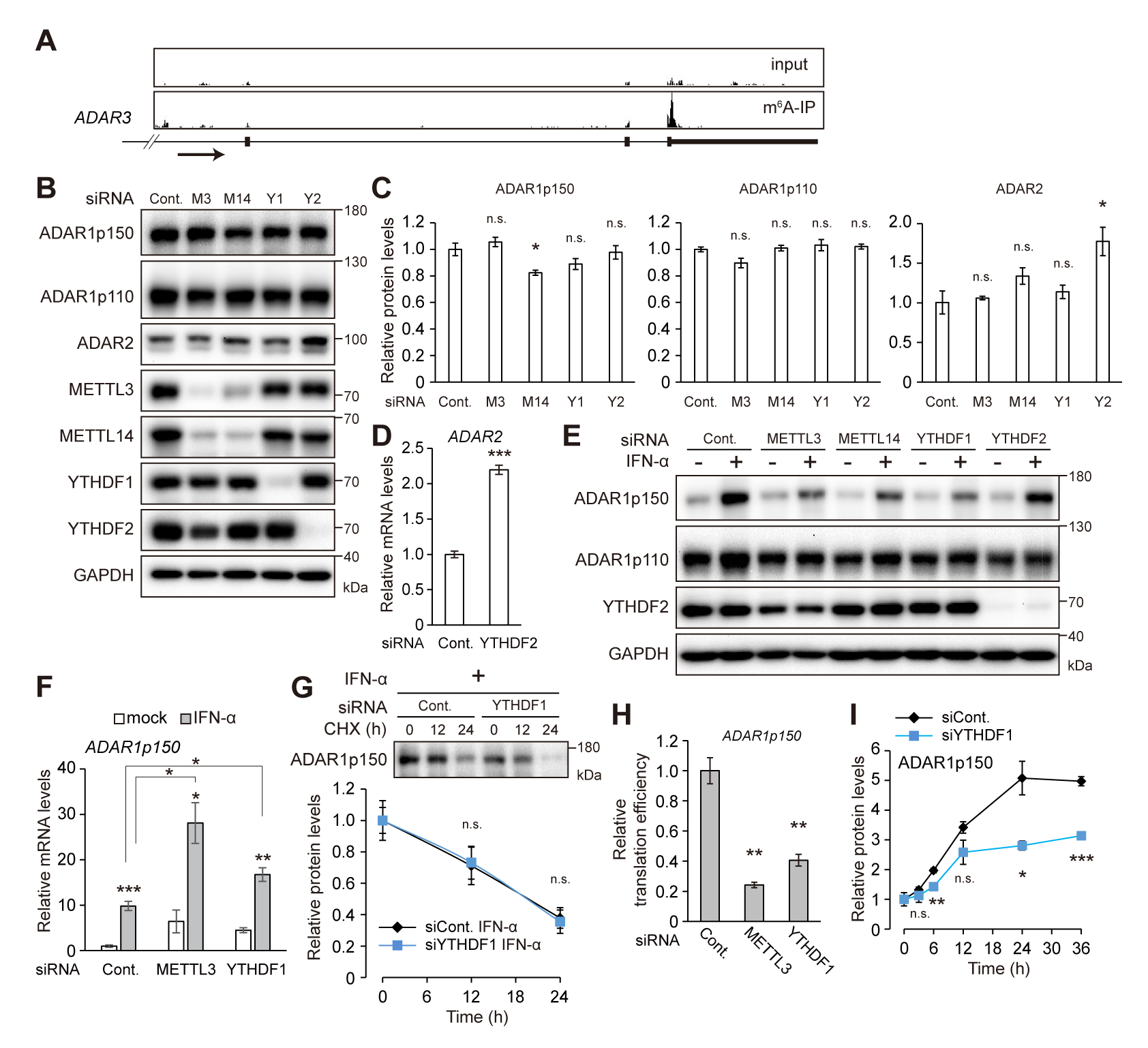

Supplement: S1 Fig — (A) m6A-seq data of the ADAR3 transcript in HepG2 cells, modified from Dominissini and colleagues [9]. An arrow indicates the transcription direction. (B, C) Immunoblot analysis showing knockdown effects of METTL3, METTL14, YTHDF1, and YTHDF2 on ADAR1p150, ADAR1p110, and ADAR2 protein expression levels in A172 cells under normal culture conditions. Immunoblot images are representative of 3 biological replicates. (D) RT-qPCR showing knockdown effect of YTHDF2 on ADAR2 mRNA. The signals were normalized to GAPDH. (E) Immunoblot analysis showing knockdown effects of METTL3, METTL14, YTHDF1, and YTHDF2 on ADAR1p150 and ADAR1p110 protein expression levels following IFN-α stimulation. Immunoblot images are representative of 3 biological replicates. (F) RT-qPCR showing knockdown effects of METTL3 and YTHDF1 on ADAR1p150 mRNA following IFN-α stimulation. The signals were normalized to GAPDH. (G) Immunoblot analysis showing time course of ADAR1p150 protein degradation in A172 cells after IFN-α stimulation. Cells were collected at 0, 12, and 24 h after the addition of CHX. ADAR1p150 protein levels at the starting point were normalized to 1. (H) Effects of METTL3 and YTHDF1 knockdown on ADAR1p150 translation efficiency in A172 cells following IFN-α stimulation (ratio of protein amounts to mRNA levels). (I) Quantification of ADAR1p150 protein expression levels from immunoblot images of Fig 1E. (C, D, F–I) Two-tailed Student t tests were performed to assess the statistical significance of differences between groups, *p < 0.05, **p < 0.01, ***p < 0.001. n = 3 for all experiments. Data are presented as the mean ± SEM. The numerical values for this figure are available in S1 Data. CHX, cycloheximide; IP, immunoprecipitation; m6A, N6-methyladenosine; n.s., not significant; RT-qPCR, quantitative reverse transcription PCR; SEM, standard error of the mean; siRNA, small interfering RNA. (TIF) [file pbio.3001292.s001.tif]

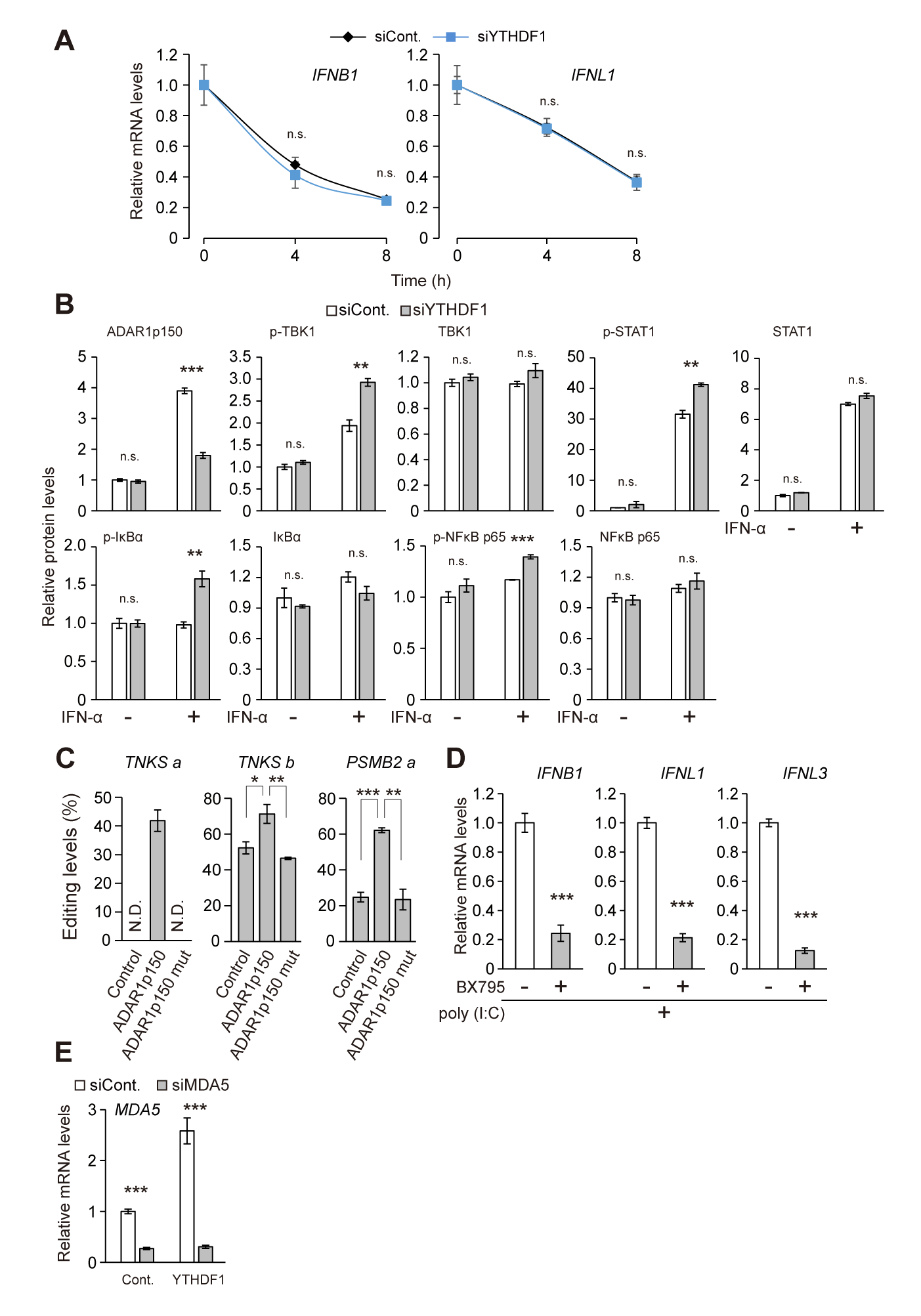

Supplement: S2 Fig — (A) Decay profiles of IFNB1 and IFNL1 mRNAs in control or YTHDF1 knockdown cells that were pretreated with IFN-α and then treated with actinomycin D. The signals were normalized to signals at the time 0 h. (B) Quantification of protein expression levels from immunoblot images of Fig 3C and 3H. (C) A-to-I RNA editing of a few selected transcripts in stable cell lines expressing control EGFP, wild-type ADAR1p150, or catalytically inactive mutant of ADAR1p150E912A, respectively. All cells were transfected with YTHDF1 siRNA and treated with IFN-α. (D) RT-qPCR showing inhibitory effects of BX795 on IFNs mRNA induction upon poly (I:C) stimulation. A172 cells that were pretreated with the BX759 inhibitor for 1 h and then transfected with poly (I:C). (E) RT-qPCR showing that knockdown efficiency of MDA5 in the samples of Fig 4D. (D, E) The signals were normalized to GAPDH. (A–E) Two-tailed Student t tests were performed to assess the statistical significance of differences between groups, *p < 0.05, **p < 0.01, ***p < 0.001, n.s. p ≧ 0.05, N.D. means not detected. n = 3 for all experiments. Data are presented as the mean ± SEM. The numerical values for this figure are available in S1 Data. A-to-I RNA editing, adenosine-to-inosine RNA editing; IFN, interferon; ISG, IFN-stimulated gene; m6A, N6-methyladenosine; N.D., not detected; n.s., not significant; RT-qPCR, quantitative reverse transcription PCR; SEM, standard error of the mean; siRNA, small interfering RNA; TBK1, TANK-binding kinase 1. (TIF) [file pbio.3001292.s002.tif]

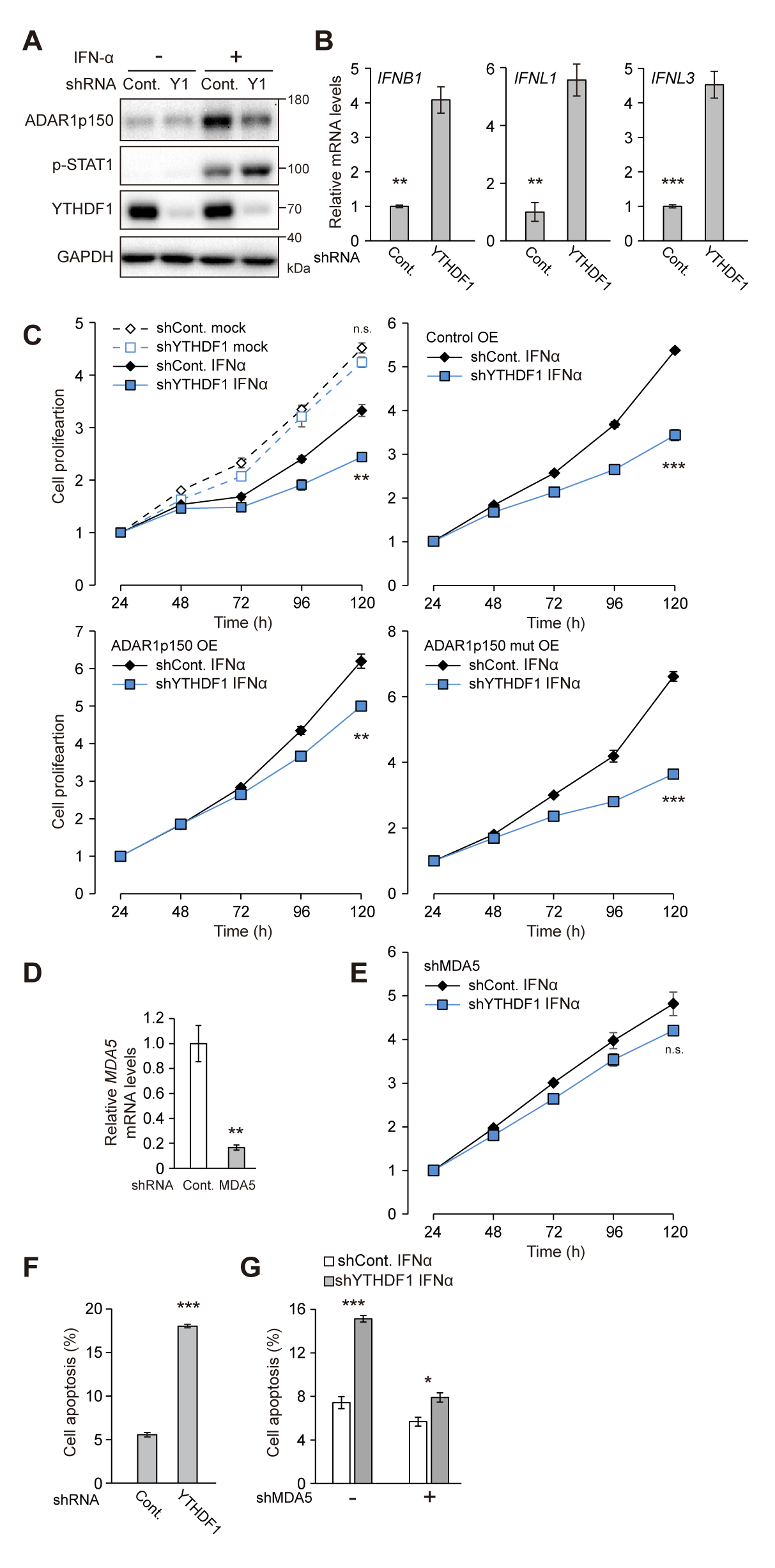

Supplement: S3 Fig — (A) Immunoblot analyzing in stable YTHDF1 knockdown cells following IFN-α stimulation. Immunoblot images are representative of 3 biological replicates. (B) RT-qPCR showing transcripts levels of IFNB1, IFNL1, and IFNL3 in stable YTHDF1 knockdown cells versus controls following IFN-α stimulation. The signals were normalized to GAPDH. (C) IFN-responsive cell proliferation rate in stable YTHDF1 knockdown and ADAR1p150 overexpressing cell lines. (D) RT-qPCR of MDA5 mRNAs in stable MDA5 knockdown A172 cells. The signals were normalized to GAPDH. (E) IFN-responsive cell proliferation rate in stable YTHDF1 and MDA5 knockdown cell lines. (F) IFN-induced apoptosis measured by TUNEL assay in stable YTHDF1 knockdown cells following IFN-α stimulation. YTHDF1 knockdown increases apoptosis. (G) IFN-induced apoptosis measured by TUNEL assay in stable YTHDF1 and MDA5 knockdown cells following IFN-α stimulation. (B–G) Two-tailed Student t tests were performed to assess the statistical significance of differences between groups, *p < 0.05, **p < 0.01, ***p < 0.001, n.s. p ≧ 0.05. n = 3 for all experiments. Data are presented as the mean ± SEM. The numerical values for this figure are available in S1 Data. IFN, interferon; n.s., not significant; RT-qPCR, quantitative reverse transcription PCR; SEM, standard error of the mean; shRNA, short hairpin RNA. (TIF) [file pbio.3001292.s003.tif]

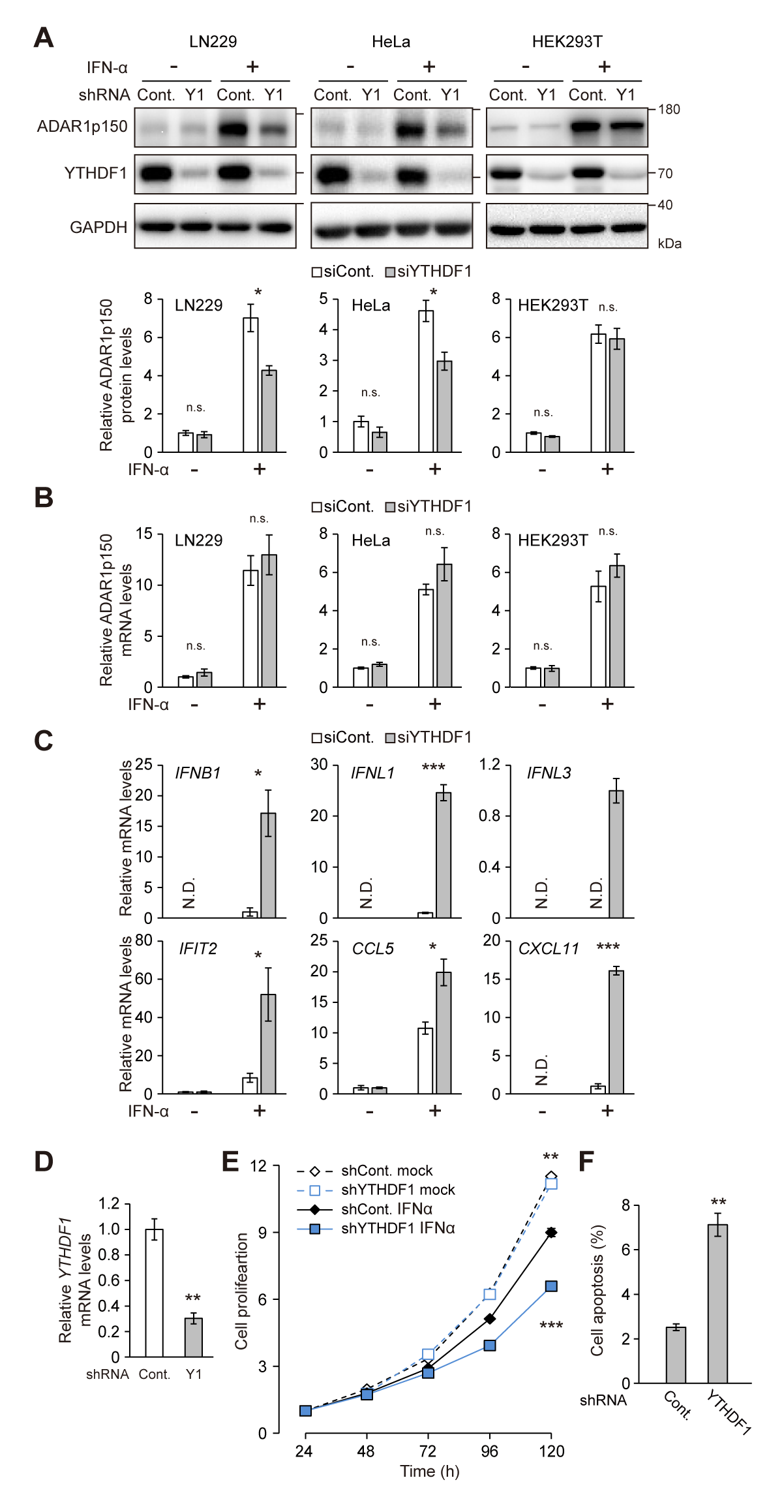

Supplement: S4 Fig — (A) Immunoblot analysis showing knockdown effects of YTHDF1 on ADAR1p150 protein expression following IFN-α stimulation in LN229, HeLa, and HEK293T cells. Immunoblot images are representative of 3 biological replicates. (B) RT-qPCR showing knockdown effects of YTHDF1 on ADAR1p150 mRNA following IFN-α stimulation in LN229, HeLa, and HEK293T cells. (C) RT-qPCR showing knockdown effect of YTHDF1 on IFN genes, ISGs, and NF-κB–inducible genes in LN229 cells. (D) RT-qPCR showing knockdown effect on YTHDF1 mRNA in stable YTHDF1 knockdown LN229 cells. (E) IFN-responsive cell proliferation rate in stable YTHDF1 knockdown LN229 cells. (F) IFN-induced apoptosis measured by TUNEL assay in stable YTHDF1 knockdown LN229 cells following IFN-α stimulation. (B–D) The signals were normalized to GAPDH. (B–F) Two-tailed Student t tests were performed to assess the statistical significance of differences between groups, *p < 0.05, **p < 0.01, ***p < 0.001, n.s. p ≧ 0.05, N.D. means not detected. n = 3 for all experiments. Data are presented as the mean ± SEM. The numerical values for this figure are available in S1 Data. IFN, interferon; ISG, IFN-stimulated gene; n.s., not significant; RT-qPCR, quantitative reverse transcription PCR; SEM, standard error of the mean; shRNA, short hairpin RNA. (TIF) [file pbio.3001292.s004.tif]

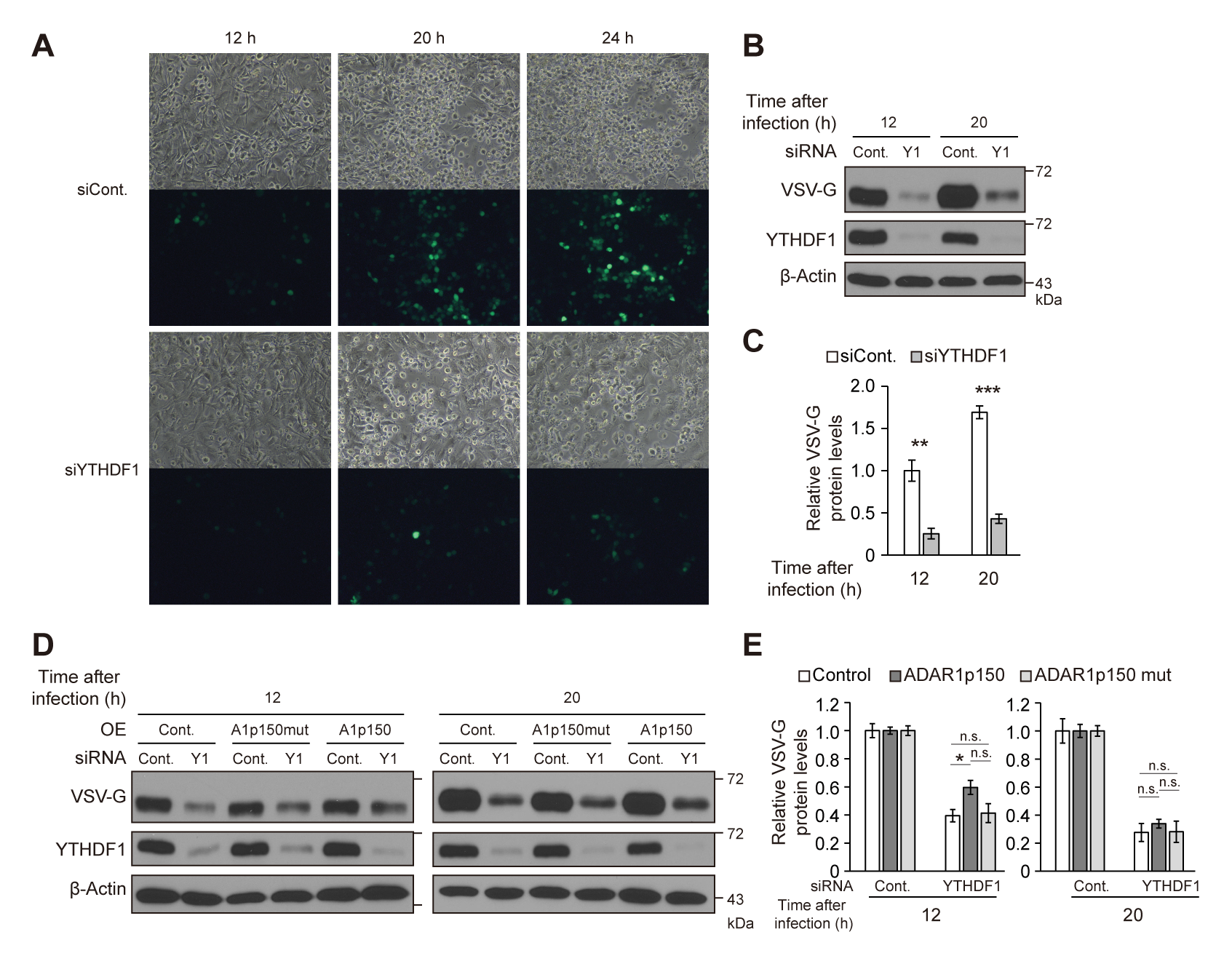

Supplement: S5 Fig — (A) YTHDF1 knockdown decreases GFP expression in rVSV-infected A172 cells. A172 cells transfected with YTHDF1 siRNA or control siRNA were infected with rVSV-GFP at an MOI of 0.1, and GFP expression was monitored at 12, 20, and 24 h postinfection. Cells are shown in the same area under bright-field and fluorescence images. (B, C) Immunoblot analysis showing significant decrease in the expression of VSV-G protein upon YTHDF1 knockdown at 12 and 20 h after rVSV-GFP infection. Immunoblot images are representative of 3 biological replicates. (D, E) Immunoblot analysis showing knockdown effect of YTHDF1 on the expression of VSV-G protein at 12 and 20 h after rVSV-GFP infection in stable cell lines expressing control EGFP, wild-type ADAR1p150, or catalytically inactive mutant of ADAR1p150E912A, respectively. Immunoblot images are representative of 3 biological replicates. The signals were normalized to control siRNA samples. (C, E) Two-tailed Student t tests were performed to assess the statistical significance of differences between groups, *p < 0.05, **p < 0.01, ***p < 0.001, n.s. p ≧ 0.05. n = 3 for all experiments. Data are presented as the mean ± SEM. The numerical values for this figure are available in S1 Data. MOI, multiplicity of infection; n.s., not significant; rVSV-GFP, recombinant GFP-expressing vesicular stomatitis virus; SEM, standard error of the mean; siRNA, small interfering RNA. (TIF) [file pbio.3001292.s005.tif]

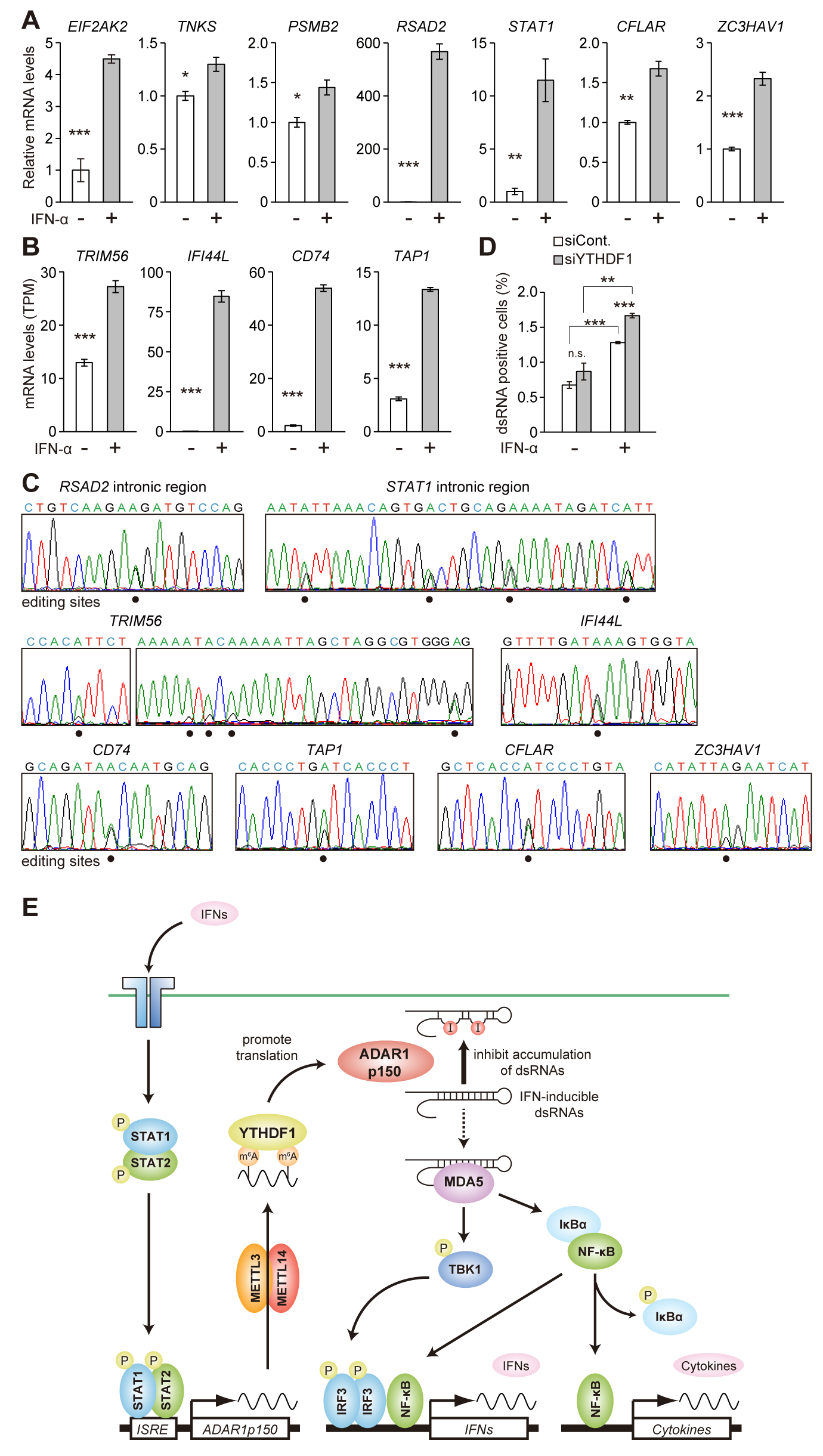

Supplement: S6 Fig — (A) RT-qPCR of EIF2AK2, TNKS, PSMB2, RSAD2, STAT1, CFLAR, and ZC3HAV1 mRNAs in A172 cells. The signals were normalized to GAPDH. (B) Expression levels of TRIM56, IFI44L, CD74, and TAP1 mRNAs in RNA-seq analysis of A172 cells. (C) Direct sequencing chromatogram showing from RT-PCR products of RSAD2, STAT1, TRIM56, IFI44L, CD74, TAP1, CFLAR, and ZC3HAV1 mRNA following IFN-α stimulation. (D) A172 cells were transfected with YTHDF1 siRNA or control siRNA and treated with IFN-α. The cells were then stained with J2 antibody and fluorescent secondary antibody and analyzed by flow cytometry. (E) A schematic model showing YTHDF1-mediated regulation of IFN responses. (A, B, D) Two-tailed Student t tests were performed to assess the statistical significance of differences between groups, *p < 0.05, **p < 0.01, ***p < 0.001. n = 3 for all experiments. Data are presented as the mean ± SEM. The numerical values for this figure are available in S1 Data. A-to-I RNA editing, adenosine-to-inosine RNA editing; dsRNA, double-stranded RNA; IFN, interferon; IRF3, IFN regulatory factor 3; m6A, N6-methyladenosine; RT-qPCR, quantitative reverse transcription PCR; SEM, standard error of the mean; siRNA, small interfering RNA; TBK1, TANK-binding kinase 1; TPM, transcripts per kilobase million. (TIF) [file pbio.3001292.s006.tif]

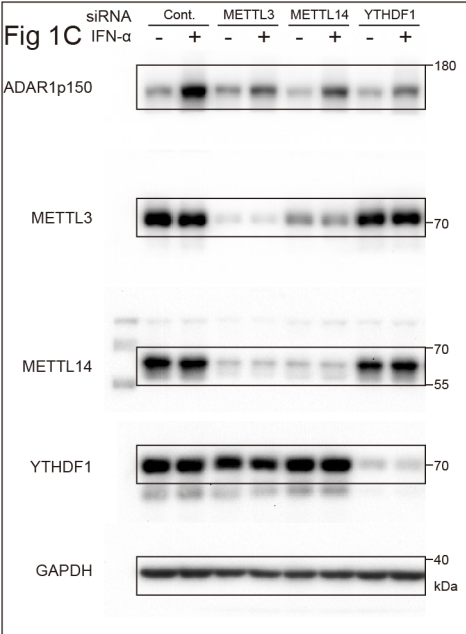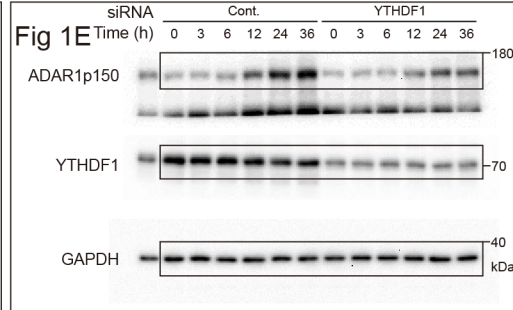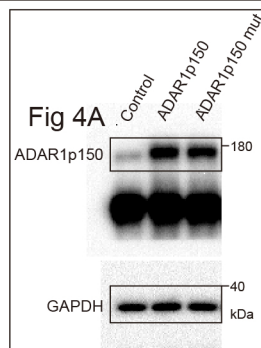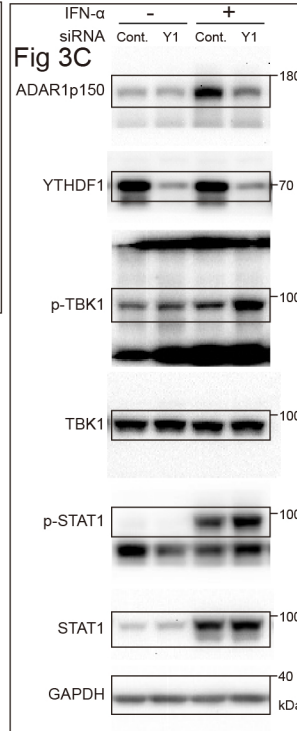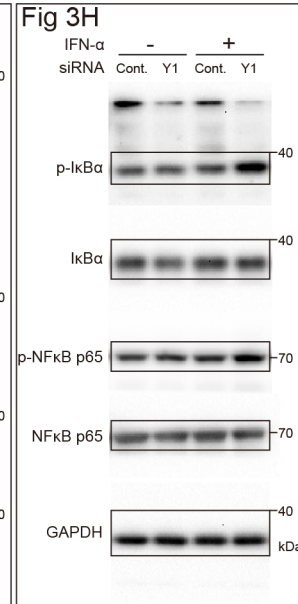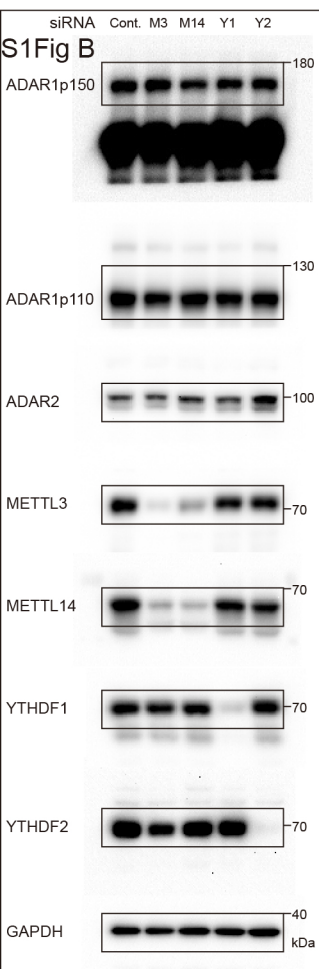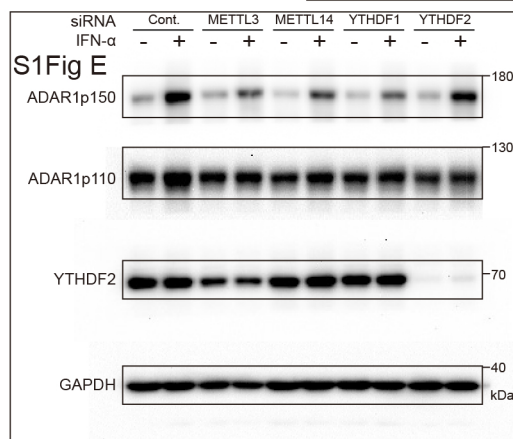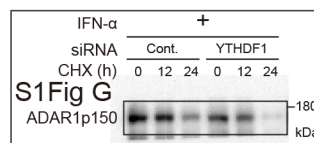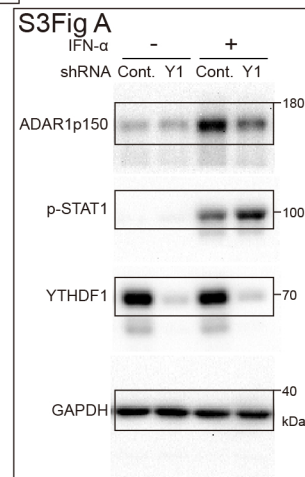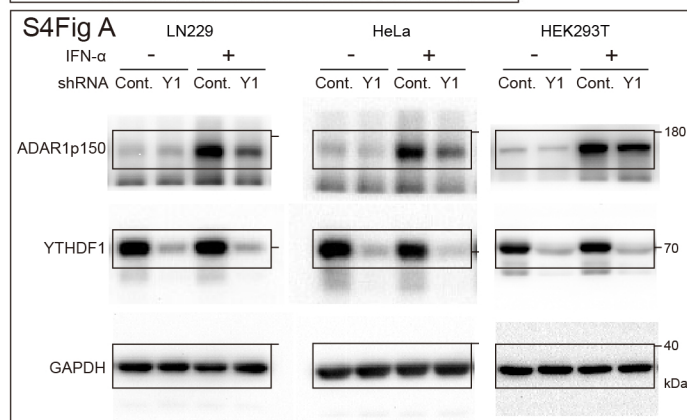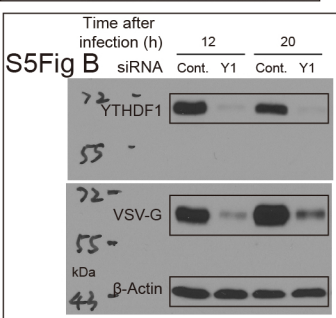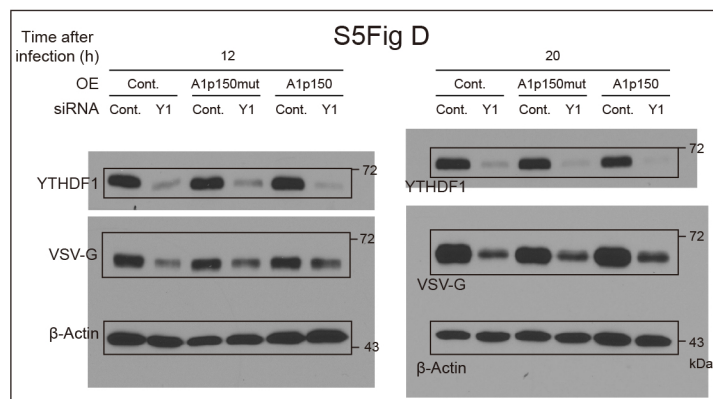

Supplement: S1 Raw Images — (PDF) [file pbio.3001292.s011.pdf]
